# Recent Advancements in the SERS-Based Detection of *E. coli*

**DOI:** 10.3390/s26020490

**Published:** 2026-01-12

**Authors:** Sarthak Saxena, Ankit Dodla, Shobha Shukla, Sumit Saxena, Bayden R. Wood

**Affiliations:** 1 IITB Monash Research Academy, Indian Institute of Technology Bombay, Mumbai 400076, Maharashtra, India; sarthak.saxena@monash.edu (S.S.); ankit.dodla@monash.edu (A.D.); 2 School of Chemistry, Monash University, Clayton, VIC 3168, Australia; 3 Department of Metallurgical Engineering and Material Science, Indian Institute of Technology Bombay, Mumbai 400076, Maharashtra, Indiasumit.saxena@iitb.ac.in (S.S.)

**Keywords:** Raman spectroscopy, SERS, EC-SERS, *E. coli*, nanoparticles, SERS substrates

## Abstract

**Highlights:**

- **Overview of SERS for *E. coli* detection**, including historical development, fundamental principles, and key factors influencing sensitivity, specificity, and reproducibility.
- **Critical evaluation of SERS substrate design**, covering fabrication strategies, nanoparticle materials, shapes, sizes, and excitation wavelength, and their impact on *E. coli* detection performance.
- **Comprehensive survey of recent advances in SERS-based *E. coli* detection**, providing a consolidated resource to help researchers select appropriate detection strategies for different applications.
- **In-depth analysis of advanced and integrated SERS approaches** for *E. coli* detection, highlighting current limitations and identifying research gaps to guide future developments.

**Abstract:**

*Escherichia coli* (*E. coli*) is a well-established indicator of faecal pollution and a potent pathogen linked to numerous gastrointestinal and systemic illnesses. Ensuring public safety requires rapid and sensitive detection methods capable of real-time, on-site deployment. Many conventional techniques are either laborious, time-intensive, costly, or require complex infrastructure, limiting their applicability in field settings. Raman spectroscopy offers label-free molecular fingerprinting; however, its inherently weak scattering signals restrict its effectiveness as a standalone technique. Surface-Enhanced Raman Spectroscopy (SERS) overcomes this limitation by exploiting plasmonic enhancement from nanostructured metallic substrates—most commonly gold, silver, copper, and aluminium. Despite the commercial availability of SERS-active substrates, challenges remain in achieving high reproducibility, long-term stability, and true field applicability, necessitating the development of integrated lab-on-chip platforms and portable, handheld Raman devices. This review critically examines recent advances in SERS-based *E. coli* detection across water and perishable food products with particular emphasis on the evolution of SERS substrate design, the incorporation of biosensing elements, and the integration of electrochemical and microfluidic systems. By contrasting conventional SERS approaches with next-generation biosensing strategies, this paper outlines pathways toward robust, real-time pathogen detection technologies suitable for both laboratory and field applications.

## 1. Introduction

**Figure 1:**
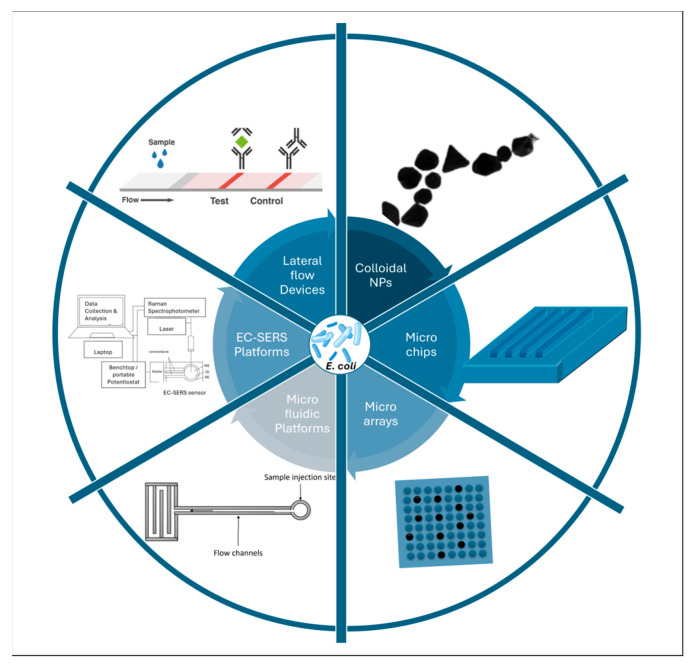
Schematic overview of SERS-based sensing platforms for *E. coli* detection.

*Escherichia coli* (*E. coli*) is a faecal coliform that is one of the major biological contaminants for clinical environments, food, and water [1]. Most types of *E. coli* are not lethal and cause diarrhoea, but a few major pathogenic *E. coli* strains, such as enterotoxigenic (ETEC), enterohemorrhagic (EHEC), and enteropathogenic (EPEC), cause severe gastrointestinal diseases, are associated with haemolytic uremic syndrome, and can lead to fatalities [2]. These contaminations pose a significant threat to public health. In middle and low-income countries, approximately one-third of foodborne disease-related deaths of children under age 5 and infants are attributed to *E. coli* contamination, resulting in a high rate of mortality and morbidity. According to the 2018 SDG 6 Synthesis Report on Water and Sanitation published by the United Nations, 4.2 billion people worldwide lack access to safe sanitation services, which further contributes to global pollution and disease burden [3]. Around 829,000 people die globally each year due to diarrhoea. Additionally, the deaths of approximately 297,000 children under the age of 5 years could be avoided through proper prevention techniques [4,5]. Diseases directly associated with climate change also spread through water, either by direct consumption or indirect consumption, such as the consumption of food irrigated with contaminated water [6]. Their transmission through environmental means, such as irrigation systems, wastewater streams, inadequate sewage treatment, and poor management, further contributes to water, sanitation, and hygiene (WASH) issues (see Figure 1).

Despite the critical importance of clean environments and safe food and water, rigorous pre-consumption testing remains challenging. Traditional microbial detection methods—whether morphological, chemical, or molecular—can take up to ten days to deliver results [7]. Direct molecular identification methods such as real-time PCR (RT-PCR) and next-generation sequencing (NGS) are highly accurate and precise, but their high cost, specialised instrumentation, and laboratory requirements limit their practicality in developing regions [8]. Whether employing culture-based, non-culture, or biochemical characterisation techniques, each approach demands substantial laboratory work, is time-consuming, and often lacks specificity. In contrast, nucleic acid-based detection methods are highly efficient but come with a significantly higher cost [9].

Spectroscopy is increasingly recognised as a rapid, low-cost, and highly effective detection technique; however, it still relies on sophisticated and expensive instrumentation [9]. Its ability to bypass culturing and minimise sample preparation makes it both fast and potentially lifesaving. In particular, modified Raman spectroscopic methods incorporating nanotechnology are emerging as powerful tools for pathogen detection [10]. Raman spectroscopy produces a unique molecular fingerprint based on the inelastic scattering of laser light by vibrational modes within the sample [11]. Traditionally used in analytical chemistry, Raman analysis has gained significant traction in biological and biomaterial research due to its molecular specificity [12]. However, spontaneous Raman scattering is inherently weak—only about one in 10^8^ photons undergo Raman scattering—restricting detection to relatively high sample concentrations (≈0.1 − 1 μM or above) [13]. To overcome these limitations, various tip-, surface-, and resonance-based Raman enhancement techniques have been developed, marking a new era in Raman spectroscopy research [14,15,16,17,18].

Surface-Enhanced Raman Spectroscopy (SERS) has a rich history evolutionary history. Since the discovery, it has been integrated with numerous complementary techniques to enhance accuracy, sensitivity, and reproducibility [19,20,21,22]. SERS magnifies the weak Raman signals in the presence of nanosized metallic structures or ions. Maximum enhancement is typically reported with silver, although significant enhancement is also observed with gold and copper [23]. Pathogen detection using SERS is challenging due to the complexities of hotspot generation, distribution, variability in nanoparticle size, and differences among bacterial species. Nonetheless, several researchers have successfully reported the detection of various Gram-positive and negative pathogenic bacteria, e.g., *E. coli*, *Staphylococcus aureus*, *Shigella flexneri*, *Bacillus subtilis*, *Salmonella typhi*, *Salmonella enteritidis*, *Pseudomonas syringae*, *Listeria monocytogenes*, etc., through SERS [24,25,26,27,28,29]. Out of all the pathogens, *E. coli* is one of the most dominant and widely available pathogens, with its presence ranging from sewage water to food items to air and various surfaces due to its high growth rate and minimal requirements for growth. Recent studies indicate that SERS has emerged as a powerful alternative detection method, offering a combination of low cost, non-destructive analysis, label-free operation, and single-cell sensitivity with high chemical specificity [22,30,31,32]. Its ability to generate detailed molecular fingerprints within minutes, without the need for extensive sample preparation, is establishing SERS as one of the leading analytical techniques. SERS is increasingly being integrated into high-throughput platforms such as microchips, microarrays, microfluidic systems, lateral-flow devices, and electrochemical sensors. The incorporation of SERS into these advanced platforms is enhancing its utility for the detection of *E. coli*, enabling shorter detection times, multiplexed analysis, stronger signal enhancement, and improved sensitivity. Collectively, these advancements provide a robust and practical solution for monitoring, managing, and preventing issues arising from bacterial contamination [33,34]. These integrated approaches have opened new realms for real-world applications in diverse domains such as food quality monitoring, clinical diagnostics, water safety, etc. [35]. Over time, these technologies are converging, with researchers integrating them with microchips, microarrays, microfluidics, lateral flow assays, and basic electrochemistry. These hybrid platforms not only improve the sensitivity, speed, and deployability but also aim to establish their use in routine monitoring programmes and mainstream applications.

This paper presents a comprehensive review of the detection of the pathogenic bacterium *E. coli* using Surface-Enhanced Raman Spectroscopy (SERS). It provides a brief overview of the history and principles of SERS, followed by a detailed compilation of SERS-based detection methods. Additionally, it delves into the characteristics of the *E. coli* pathogen, explores its detection techniques, and highlights emerging advancements in SERS-based *E. coli* detection.

## 2. History, Introduction, Important Factors in SERS-Based Research

Raman spectroscopy utilises the Raman effect, which was discovered by an Indian physicist, Dr Chandrashekhara Venkata Raman, in 1928 [18]. It describes the change in frequency of scattered light after interaction with an analyte molecule [18,36].

### 2.1. History

The SERS phenomenon was first discovered by independent studies of Albrecht and Creighton and Van Duyne and Jeanmaire in 1977 and was reported earlier by Fleischmann and colleagues in 1974 [37,38]. After these studies, several other independent researchers also reported similar phenomena [39,40]. Jeanmaire et al. reported a remarkable enhancement (10^6^-fold) in the Raman signals of pyridine on the electrochemically roughened silver electrode surface. Jeanmaire also reported that the enhancement of some peaks that were not present in the normal Raman spectrum at low laser power was observed, opening the realms of surface- and resonance-based Raman spectroscopy [38]. Creighton et al. compared the enhancement on silver and gold surfaces and concluded that the degree of signal enhancement depends upon the excitation wavelength, Mie scattering, and particle size. The study demonstrated that surface plasmon oscillations are responsible for Raman signal enhancement. Scattering profiles on gold and silver surfaces were found to be similar, and enhancement was predicted to be maximum on coarsely roughened surfaces compared with small particles [41]. These studies stimulated numerous investigations into molecular enhancement phenomena, firmly established SERS as a distinct effect, and directly linked it to plasmonically active metallic substrates [42,43,44]. In addition to the pioneering experiments by Albrecht and Creighton and Jeanmaire and Van Duyne in 1977, the SERS field expanded rapidly during the late 1970s and early 1980s. After the initial demonstrations, Moskovits proposed in 1978 that localised surface plasmon resonance (LSPR) on roughened metal surfaces underlies much of the Raman enhancement through collective oscillations of conduction electrons [45]. Soon after, Creighton and Albrecht extended the phenomenon to colloidal silver and gold nanoparticles, demonstrating that such nanostructures could reproducibly generate strong enhancement effects [45,46]. Over the subsequent decade, the community posited a dual-mechanism model, combining electromagnetic enhancement arising from plasmon resonances with a chemical (or charge-transfer) contribution between the molecule and the metal, to explain observed spectral shifts, peak intensity variations, and voltage-dependent behaviour in electrochemical SERS [47,48,49]. These conceptual advances established SERS not only as a powerful analytical technique but as a valuable platform for investigating surface physics and nanoscience.

### 2.2. SERS

**Figure 2:**
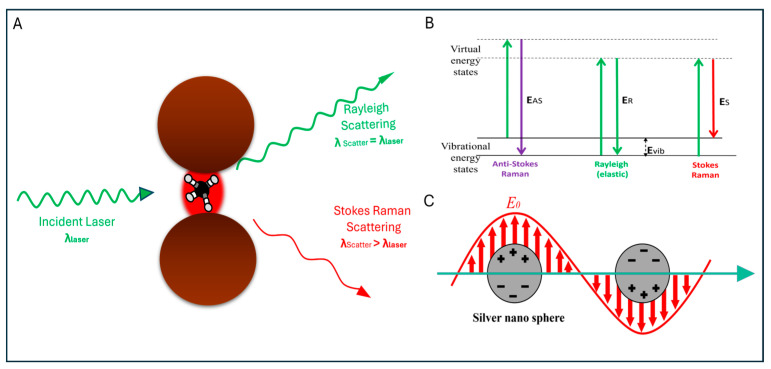
(**A**) Raman and Rayleigh scattering of a laser by the analyte molecule kept under a hotspot generated by two metallic nanoparticles; (**B**) the quantum energy diagram of the analyte molecule showing the energy transitions for the Raman and Rayleigh scattering; (**C**) localised surface plasmon resonance around the metal nanoparticles. Figures are adapted from [54] and reproduced with permission from the American Chemical Society, Copyright © 2022.

Surface-Enhanced Raman Spectroscopy (SERS) has been recognised as an effective detection tool due to its higher molecular specificity, as demonstrated over many years [50]. It enhances the weak Raman signals of specimens up to approximately 10^5^–10^15^-fold in the presence of surface plasmons generated by silver, gold, or copper nanostructures [51]. The surface cross-section and morphology significantly affects the effectiveness of SERS signals. Since Raman signals are inherently weak, Surface-Enhanced Raman Spectroscopy provides a larger effective surface area and intense local electromagnetic fields that are strong enough to detect Raman scattering of molecules [42] (Figure 2). Due to the higher sensitivity and enhanced spectroscopic insight, SERS is an ideal technique for analysing very low concentrations of analytes [51].

The major mechanisms responsible for the SERS effect are electromagnetic and chemical enhancement. Although other theories have been proposed, these two are widely accepted and explored to date [36].

Kudelski et al. concluded that enhancement in Raman signals is primarily attributed to the electromagnetic mechanism (increase in the electric field on the metal surface), while a smaller part of it arises from the charge-transfer because it resembles the normal Raman scattering observed in metal–ligand complexes [52]. The electromagnetic enhancement theory explains the SERS effect through interactions between electromagnetic waves and metallic nanostructures. When an electromagnetic wave interacts with a metallic surface, it induces changes in the electric field. The roughness of the surface can lead to the excitation of localised surface plasmons, resulting in a significant enhancement of the electromagnetic field around the molecule on the surface. This enhancement in the intensities of both the incident and scattered fields (relative to the wavelength of the incident beam) is believed to contribute to the significant amplification of Raman signals [53]. This phenomenon is referred to as the electromagnetic (EM) mechanism of SERS and plays a crucial role in understanding the SERS effect.

The electromagnetic (EM) mechanism is considered the dominant contribution and is often referred to as the “first-layer effect,” as it explains the direct interaction between the metal surface and the analyte molecule [36]. In contrast, chemical enhancement theory is considered an explanation for the electron transfer between the analyte and the plasmonic surface [55]. However, it involves several other sub-mechanisms as well. The chemical enhancement theory explains the increase in SERS signals through adsorption processes. The analyte molecules adsorb on roughened metallic surfaces, causing a change in the electronic states of the adsorbed species.

This may happen as a result of chemisorption or bond formation between the analyte molecule and the metallic surface [36]. John and George proposed three distinct mechanisms for chemical enhancement in SERS. The first mechanism suggests that Raman signal enhancement occurs due to the interaction between the analyte molecule and the metallic surface in the ground state. The second mechanism attributes the enhancement to resonance effects, where the excitation wavelength causes molecular transitions that result in resonance, thereby amplifying the Raman signals. The third mechanism explains that Raman signal enhancement arises from charge-transfer transitions between the analyte molecule and the metallic surface, which also induce resonance effects [51].

### 2.3. Important Factors in SERS-Based Research

The enhancement of SERS signals is governed by multiple interrelated factors. Among the most critical are the composition and morphology of metal nanostructures, the nature of the supporting substrates, and the properties of the excitation laser. This section provides a detailed examination of how each of these parameters contributes to the amplification of Raman signals.

#### 2.3.1. Nanoparticle Characteristics and Their Impact on Signals

The characteristics of nanoparticles influence the SERS signal. The composition and morphology of nanoparticles play an important role in generating SERS spectra. Metallic nanostructures, including silver [56,57], gold [58,59], and copper [60,61] nanoparticles (NPs), are widely utilised in SERS applications due to their strong localised surface plasmon resonance (LSPR) within the visible to near-infrared spectral range [62]. Additionally, other metals such as aluminium [63], platinum [64], and palladium [64,65]-based nanostructures serve as plasmonic materials for SERS, particularly in the ultraviolet spectral range. Silver nanostructures generally yield stronger SERS enhancements than gold or copper nanostructures. Gold-based nanostructures are more widely employed due to their inert nature, superior chemical stability, ease of functionalisation, and versatility in forming a wide range of morphologies [66,67]. SERS signal enhancement generally depends on the laser wavelength. Silver nanostructures show higher enhancement when used with green lasers. In contrast, gold nanostructures, better enhancement comes with red lasers [68,69,70].

SERS enhancement can occur on single NPs due to the local electric-field amplification. Significantly higher enhancements (EFs ≈ 10^5^–10^6^) can be achieved by creating nanometre-sized gaps between the metal nanostructures known as hotspots [66,71,72]. Hotspots arise at nanoparticle junctions or on flat plasmonic surfaces, where the local field strengths are roughly inversely proportional to the gap dimensions and are shaped by the geometry [47,49]. Hotspots occurring in the 2–10 nm range can be explained using classical electromagnetism by considering the frequency-dependent dielectric properties of the material in the nanostructure and neglecting non-local effects [49].

Hybrid nanoparticles are advanced SERS substrates that fuse plasmonic materials with semiconductors, magnetic components, carbon-based structures, or dielectric coatings to achieve synergistic enhancements in sensitivity and functionality. Examples include Au–Ag bimetallic NPs [73,74,75], magnetic–plasmonic hybrids [76], core–shell metal–dielectric hybrids resulting in Shell-Isolated Nanoparticle-Enhanced Raman Spectroscopy (SHINERS) [77], and metal–carbon nanostructures [78,79].

The size of nanoparticles significantly influences the extinction coefficient and position of the LSPR peak, thereby affecting the SERS enhancement factor (EF). For spherical nanoparticles, an effective size range of 5–100 nm is typically observed, with larger diameters correlating with red-shifted LSPR peaks and increased EFs [80,81]. Among gold and silver nanoparticles, sizes around 50–60 nm have consistently shown optimal SERS performance across multiple studies, aligning well with the commonly used excitation wavelengths [82,83,84,85,86]. Below ~3 nm, spherical NPs lose plasmonic behaviour due to strong surface damping, while particles larger than 100 nm exhibit broadened LSPR peaks caused by radiative damping of dipolar oscillations [87,88,89].

The morphology of nanoparticle structures plays a critical role in determining SERS signal intensity, as it directly influences the distribution and strength of localised electromagnetic fields. A wide range of nanostructures has been investigated for their SERS performance, including nanotriangles [90,91,92], nanorods [93,94], nanostars [95,96], nanowires [97,98], nanoflowers [99,100], nanopillars [101,102], nanobowls [103], nanourchins [104,105], nanopancakes [106], nanoballoons [107], NP-coated microneedles [29], NP capsules [108], and NP sandwiches [57,109]. Both experimental and theoretical studies have shown that anisotropic nanostructures such as nanostars, nanorods, and nanotriangles generate stronger localised electromagnetic fields at their tips and edges. These structural features result in significantly higher SERS enhancement compared to spherical nanoparticles [66,78,110,111,112].

#### 2.3.2. Laser Characteristics

The laser is a critical factor in SERS, as its characteristics, particularly wavelength, power, and polarisation, directly influence the excitation of LSPR in plasmonic nanoparticles. Typically, the excitation wavelength is chosen to match the LSPR peak of the nanoparticles, as this alignment maximises the electromagnetic enhancement [69]. To achieve this, the extinction spectrum of the nanostructure is first measured using UV–Vis spectroscopy to identify the optimal LSPR wavelength. In SERS, the excitation wavelength is usually slightly blue-shifted from the LSPR peak to balance excitation and emission efficiencies and maximise overall enhancement [53].

SERS enhancement factors scale with the fourth power of the local electromagnetic field, with substantially greater amplification when the excitation wavelength is resonant with the plasmonic substrate’s LSPR, thereby offering practical advantages for boosting signal intensity [113,114,115]. UV and deep-UV excitations have been explored, particularly with plasmonic nanostructures based on Al, Pt, and Pd [63,64,116,117,118]. However, shorter-wavelength excitation often leads to photobleaching of biomolecules, which limits practical use. Most SERS experiments employ excitation wavelengths ranging from the blue region to the NIR [119]. Gold NPs, whose LSPR peaks can be tuned into the NIR depending on size and morphology, are widely used in biological SERS due to their biocompatibility, minimal autofluorescence, and ability to penetrate deeper into tissues [120,121].

In SERS, incident laser power must be carefully tuned to prevent local heating and photodissociation at hotspots, which can introduce carbonaceous artefacts and causes signal decay [122,123,124]. Mathematical models show that higher initial power and longer exposure times lead to dramatic drops in SERS intensity [125], while lower laser intensities yield higher enhancement factors (up to ~10^10^) before photobleaching occurs. Best practices, therefore, employ minimal laser power, short integration times, measurements in solution or by scanning dry substrates, and a multiple-point collection strategy where each spot is irradiated for under 10 μs across non-overlapping sites to maintain signal integrity [125,126]. Standardisation of laser power, spot size, and alignment is achieved through ratiometric SERS measurements employing internal standards, as previously reviewed [127].

The polarisation of the incident laser significantly influences SERS enhancement, with the highest enhancement factors typically observed at the junctions of coupled nanoparticles when the electric field is aligned across the gap [128,129,130]. Anisotropic nanostructures, such as nanorod arrays and nanocubes, exhibit a strong dependence on both the polarisation and their angle of incidence, necessitating precise sample orientation to maximise hotspot excitation and signal intensity [131,132,133].

#### 2.3.3. Substrate Characteristics

Substrates play a crucial role in SERS analysis. Their effectiveness is influenced by several factors, including the presence of substrate plasmons, Raman activity of substrate materials, the location of hotspots, substrate crystallinity, the lifetime and synthesis of substrates, and their overall suitability for various detection applications [99,134,135,136,137]. A wide range of materials have been used to develop SERS substrates. These include glass slides, coverslips, calcium fluoride, and aluminium-coated glass slides, which are mainly used as supporting SERS substrates. Several metal oxides such as titania (TiO_2_) [135], ferric oxide (Fe_3_O_4_) [25], copper oxide (Cu_2_O) [136], and silicon dioxide (SiO_2_) [138], along with polymers like polydimethylsiloxane (PDMS) [139] and polyvinylpyrrolidone (PVP) [140,141], along with other materials including chitosan [58], methyl methacrylate (C_5_H_8_O_2_) [142], calcium oxide (CaO) [143], tin (IV) oxide (SnO_2_) [144], zinc oxide (ZnO) [56], and various flexible surfaces have also been explored as SERS substrates.

Substrate selection for SERS detection has evolved. Modified SERS substrates fabricated through various emerging techniques, e.g., green synthesis [145], bioprinting [146], nanoimprinting [147], etching [148], sputtering [149], solvent casting [150], doping, 3D printing [151]-often report higher enhancement factors and shorter accumulation times at low laser power. There are three major methods of substrate fabrication and application in SERS (Table 1) for *E. coli* detection.

Ranging from the direct usage of colloidal solutions of plasmonic nanoparticles to advanced plasmonic microstructures or nanoparticle-coated microstructures, these engineered structures provide the uniform distribution of hotspots but also offer high Enhancement Factors (EFs) and effective Limits of Detection (LODs) values. The various substrate types and configurations used for SERS detection, as summarised in Table 1, are briefly discussed here.

##### Colloidal Nanoparticles and Microstructure Surfaces

Colloidal nanoparticles are among the materials most extensively employed as a substrate in SERS applications, primarily due to their ease of synthesis, broad availability, and moderate signal enhancement capabilities. Typically composed of noble metals such as gold or silver, these plasmonic nanostructures can be synthesised via well-established chemical reduction methods and are often functionalised to improve biocompatibility and target specificity. In SERS-based detection, colloidal nanoparticles, their composites, or hybrid plasmonic assemblies are directly mixed with the analyte solution, followed by drop-casting onto solid supports such as glass, calcium fluoride, or aluminium slides. In the case of plasmonic microstructures, the analyte is directly drop-cast onto the plasmonic microstructure surface. These prepared substrates are then subjected to Raman spectral analysis. Another technique utilises solid microstructures coated with the plasmonic colloidal nanoparticles. Analyte molecules are drop-cast onto these coated surfaces for Raman analysis.

Despite their practical advantages, colloidal systems are often limited by issues of chemical and colloidal instability, which can adversely affect signal consistency and reproducibility. Nonetheless, controlled aggregation of colloidal nanoparticles has been shown to significantly amplify Raman signals by increasing the density of electromagnetic “hotspots” [108]. This delicate balance between nanoparticle stability and signal amplification remains a core challenge in colloidal SERS research, particularly when reproducibility is essential under fluctuating environmental conditions. However, advancements in nanofabrication technologies have ushered in a new class of plasmonic microstructure substrates that offer significantly higher and more consistent enhancement factors. Techniques such as electron-beam lithography, nanoimprint lithography, sputtering, and hot-pressing have enabled the precise engineering of surface morphologies at the nanoscale, leading to more uniform hotspot distribution and improved signal reproducibility [147,152]. These structured substrates are increasingly favoured for applications demanding high sensitivity, durability, and integration into portable or lab-on-chip platforms.

##### Porous Materials and Commercially Available Substrates

The surface area of a SERS substrate is the critical determinant of signal intensity and detection sensitivity. An increased surface area offers more active adsorption sites, which promote the generation of a dense network of electromagnetic hotspots—localised regions where the Raman scattering signal is significantly amplified [153]. To exploit this advantage, porous materials are widely utilised as foundational scaffolds, subsequently functionalised with plasmonic nanoparticles or metal ions to enhance their SERS activity.

Materials such as graphene, carbon nanowires, metal–organic frameworks (MOFs), and commonly used porous structures, including SiO_2_, TiO_2_, Al_2_O_3_, and porous silicon, have attracted considerable interest due to their high porosity, chemical tunability, and capacity to support uniform nanoparticle distribution [153,154,155]. The commercial availability of many of these substrates further streamlines their application, making the preparation process more accessible and time-efficient. When integrated with noble metal nanostructures such as silver or gold, these porous platforms exhibit moderate to high sensitivity, excellent reproducibility, and uniform signal distribution across the surface [135,154]. Their versatility, ease of synthesis, and compatibility with a variety of analytes contribute to their broad appeal in the SERS community [155].

Functionally, these porous nanocomposites are typically mixed with the target analyte and drop-cast onto solid substrates, such as glass or calcium fluoride slides, for Raman analysis, similar to colloidal approaches. However, enhanced control over nanoparticle immobilisation and hotspot distribution offered by porous materials often leads to more stable and reproducible signals, as well as higher enhancement factors. These properties make them particularly suitable for real-world applications that require consistent, high-performance detection across diverse conditions.

##### Flexible Substrates

Flexible substrates are increasingly gaining prominence in SERS research due to their superior conformability to irregular surfaces, compatibility with non-invasive and on-site sampling techniques, potential for reusability, and low fabrication costs. Their mechanical flexibility and enhanced biocompatibility render them particularly suitable for point-of-care diagnostics and contamination-free sampling from complex biological surfaces, such as human skin, wounds, and soft tissues, as well as from irregular food matrices and environmental surfaces [155]. However, despite their advantages, many studies have reported relatively low enhancement factors when using flexible substrates alone.

To overcome this limitation, recent research efforts have focused on coupling flexible platforms with microstructure architecture, electrochemical modulation, and nanoparticle composite coatings to improve signal amplification. Various flexible materials—such as filter paper, tobacco paper, cellulose-based paper, textiles, and elastomeric polymers like polydimethylsiloxane (PDMS)—have been coated with plasmonic nanoparticles to serve as cost-effective and efficient SERS-active platforms [137,156,157]. For instance, Wang et al. developed a wearable SERS substrate integrated onto gloves for the detection of glucose in human sweat, achieving a limit of detection (LOD) of 1.68 × 10^−7^ M [158]. Similarly, Zhou et al. demonstrated the detection of pathogenic *Staphylococcus aureus* and *Shigella flexneri* using gold nanoparticle-coated tobacco paper [24].

The fabrication of flexible SERS substrates via template moulding and nanoimprint lithography has shown promising results in enhancing Raman signal intensity and uniformity. For example, Colnita et al. employed nanoimprint lithography on PDMS to synthesise flexible SERS substrates and reported an LOD of 10 pM [147]. Such improvements are often attributed to the better hotspot uniformity and ease of surface modification afforded by flexible materials.

Despite these advancements, the majority of reported flexible substrates remain limited to paper-based or PDMS-derived systems [95]. This indicates a considerable research gap in the development of novel, reproducible, and high-performance flexible SERS materials. Future work must explore advanced composites and hybrid fabrication methods to unlock the full potential of flexible SERS substrates in clinical diagnostics, environmental monitoring, and food safety applications.

## 3. Pathogens and Dominance of *E. coli*

Bacterial cells are highly sensitive to various chemical compounds, lasers, and metals like copper and silver. Due to higher SERS response, AgNPs are extensively used for SERS. However, due to its bactericidal effect, cells can die during analysis. A few researchers explored this aspect and concluded that it can be used for bacterial inactivation, too. Pathogenic bacterial species are the most explored biomolecules for SERS detection. A wide range of pathogenic bacteria-including Gram-positive, Gram-negative, acid-fast, non-fermentative, non-enterobacterales, spore-forming, non-spore-forming, and either cocci or rod-shaped-have been analysed using SERS detection. The most commonly detected and problematic bacterial species, e.g., *B. subtilis*, *S. typhi*, *Pseudomonas aeruginosa*, *S. aureus*, and *E. coli*, along with moderately or less harmful species including *Acinetobacter baumannii*, *Vibrio anguillarum*, *Vibrio harveyi*, *Edwardsiella piscicida*, *Pseudomonas plecoglossicida*, *Staphylococcus hominis*, *Burkholderia multivorans*, *Haemophilus influenzae*, *Achromobacter xylosoxidans*, *Neisseria meningitidis*, and *Vibrio parahaemolyticus*, have been detected using SERS with high enhancement factors and efficiency. *E. coli* is the most common and widely researched pathogenic bacterium due to its fast growth rate, low nutrient requirements, easy handling, and relatively simple working conditions. It is a non-spore-forming, Gram-negative, and rod-shaped bacterium that grows easily in any standard media [159]. Due to its infectious nature and harmful impact on humans, it has become an area of increasing interest.

## 4. *E. coli* Detection

**Figure 3:**
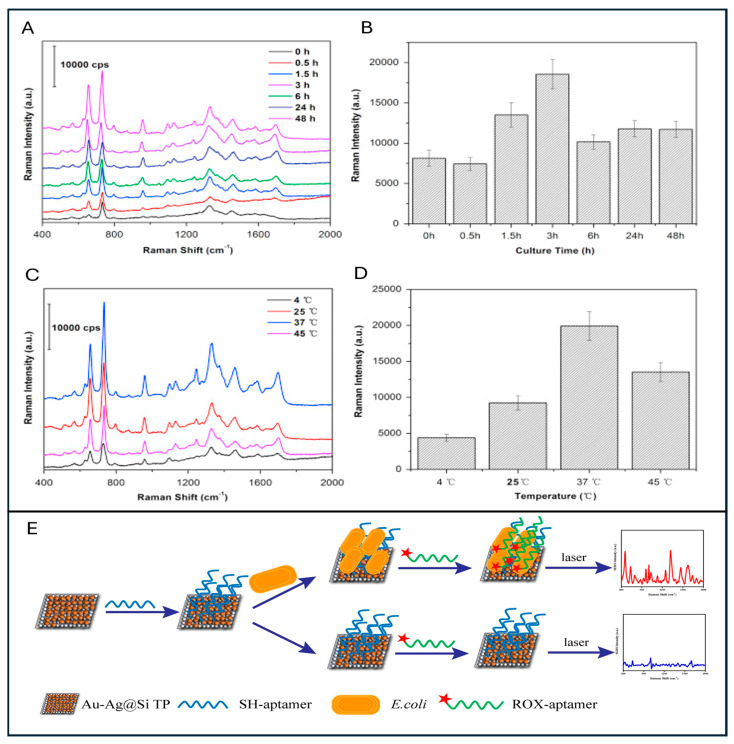
(**A**) The SERS spectra and (**B**) SERS intensities of *E. coli* DSM 1116 at different incubation duration times. (**C**) The SERS spectra and (**D**) SERS intensities of *E. coli* DSM 1116 at different incubation temperatures. Adapted from [170] and reproduced with the permission of Copyright © 2015 Elsevier. (**E**) Schematic diagram representing the aptamer-loaded sandwich method for the SERS detection of *E. coli*. Adapted from [171] and reproduced with the permission of Copyright © 2024 Elsevier.

The journey of SERS-based *E. coli* detection began in earnest with the pioneering work of Hassan et al. in 1998, marking the first documented instance of Raman signal enhancement from this pathogen [35]. Prior to this, foundational studies on DNA bases [160], nucleic acids [161], pyridine [162], membrane proteins [163] were ongoing. Later, Nabiev et al. conducted a comprehensive study and demonstrated the potential of SERS in detecting biomolecular components such as DNA, amino acids, peptides, membrane proteins, and protein–pigment complexes, setting the stage for understanding the molecular underpinnings of SERS signal enhancement [164]. Between 1990 and 2000, many independent investigations expanded this foundational knowledge, focusing on the SERS detection of key biological molecules like nicotinamide adenine dinucleotide (NAD), flavin adenine dinucleotide (FAD), and specific amino acids [165,166,167,168,169]. Figure 3 illustrates the relation between the Raman spectra, incubation time, temperature, and Raman intensities.

Notably, the earliest attempt to apply SERS for whole-cell bacterial detection was undertaken by Alexander et al., who analysed spores of *Bacillus stearothermophilus*, noting stark differences between conventional Raman and SERS spectra upon interaction with gold nanoparticles [172]. Furthering this line of inquiry, Zeiri and Efrima, in 2004, offered a comparative analysis of gold and silver nanoparticles in *E. coli* SERS, observing that gold provided better spectral resolution in the 1320–1340 cm^−1^ range, whereas silver offered distinct spectral features below 1000 cm^−1^ [173,174].

Around the same time, Sengupta et al. emphasised the role of physicochemical factors—such as incubation time, solution pH, bacterial density, and species specificity—in shaping the SERS response, underlining the technique’s sensitivity not only to molecular content but also to experimental conditions [175]. Typical *E. coli* Raman spectra exhibit numerous distinct vibrational bands. The detailed assignments of these bands, along with their corresponding molecular origins, are summarised in Table 2.

From a spectral standpoint, *E. coli* displays a rich biochemical fingerprint, with key Raman bands arising from cellular components such as nucleic acids, proteins, lipids, and polysaccharides. Among the most diagnostically relevant bands, the 732–738 cm^−1^ peak is typically assigned to the ring-breathing modes of adenine [59,185,186,187,188], while 1000–1004 cm^−1^ corresponds to phenylalanine, reflecting protein presence [11,13,176,182,186,197,203,205]. Additional enhanced bands around 1240–1300 cm^−1^ (amide III and CH bending) [11,108,137,146,179,183,184,186,193,200,201,207,208,209], 1450 cm^−1^ (CH_2_ bending of lipids) [146,178,184,187,189,209], and 1655 cm^−1^ (amide I vibration) further enrich the spectrum, providing detailed insight into the bacterium’s physiological and structural state.

In 2006, Efrima et al. made a compelling observation that the SERS spectral signature of *E. coli* shares remarkable similarity with FAD, suggesting that the redox-active biomolecule may serve as a key Raman-active component in *E. coli*’s spectral profile [216]. When interfaced with metallic nanostructures, these spectral features become significantly amplified, enabling reliable detection of *E. coli* in challenging sample matrices such as drinking water, fresh produce, and processed foods [217]. Moreover, subtle shifts in band intensity or position can reveal strain-level differences or stress responses, thus empowering both qualitative identification and quantitative assessment [185].

Numerous studies have explored the detection of *E. coli* using SERS, with the direct application of gold or silver nanoparticles (Au/Ag NPs) via drop-casting being among the most frequently employed techniques [185,186,193,197]. While this approach offers simplicity and accessibility, it often demands substantial preparation time, an aspect at odds with the inherently rapid nature desired of SERS-based methods. For instance, Jayan et al. demonstrated ex situ SERS detection of *E. coli* in poultry wash water, reporting a synthesis time of approximately 120 min for the nanostructures involved [180].

In recent years, the landscape of SERS detection has expanded significantly. Table 3 compiles several recent studies on *E. coli* detection using different types of substrates and their effectiveness in terms of the enhancement factor or LOD, along with the acquisition parameters. Beyond simple metallic nanoparticles, composite nanostructures, particularly Ag-Au nanoparticle hybrids, have gained attention due to their superior enhancement factors. Variations in nanoparticle shape, composite configuration, and deposition method have been shown to critically influence the sensitivity and reproducibility of *E. coli* detection. Among these, self-assembly and drop-casting remain the most straightforward and rapid methods, requiring minimal or no substrate fabrication [180,185,197]. However, studies suggest that pre-coating substrates with nanoparticles—especially through techniques such as layer-by-layer assembly—can yield markedly higher enhancement factors, with values reaching up to 10^9^, as reported by Li et al. [108]. The progression from colloidal methods to structured substrate-based approaches has introduced the use of both rigid and flexible platforms for SERS detection. Techniques like photolithography, electron-beam lithography, soft imprinting, and micromoulding are now being utilised to fabricate substrates with precisely engineered nanostructures. These engineered surfaces not only improve sensitivity but have also enabled detection limits as low as 0.5 CFU/mL [29,139,213].

However, many of these fabrication methods are time-intensive, which challenges the goal of rapid, point-of-care diagnostics. To address this, current efforts focus on maximising hotspot density, optimising nanoparticle concentration, and designing reproducible, high-surface-area architectures to reduce accumulation time while maintaining or enhancing signal strength [201]. Altogether, SERS presents a compelling platform for *E. coli* detection that is rapid, label-free, and capable of single-cell sensitivity. As it continues to evolve with advances in nanomaterials, substrate design, and portable Raman instrumentation, SERS holds great promise for enhancing surveillance efforts in food safety, environmental monitoring, and clinical diagnostics [226].

Rapid detection of pathogens in water is a major challenge for global safety. Clean and safe water is the priority of the World Bank and UNICEF. Out of the 26 sustainable development goals (SDGs), around eight are related to water, which highlights its importance. In current research, for the rapid, low-cost detection of pathogens, mainly *E. coli* nanoparticle-coated flexible cellulosic films have been synthesised. To enhance detection reproducibility, these films are cast with microstructures on their surface through a template moulding technique. Due to the cellulose polymer, these films are biocompatible, and their flexibility makes them useful in other areas of research, such as food, biomedicine, etc. This study proposes the fabrication of lateral-flow-based *E. coli* detection, identification, and differentiation films for on-site sampling and SERS detection.

## 5. Recent Advances in *E. coli* Detection

The detection of *E. coli* using SERS has emerged as a well-established and rapidly evolving field. Since its initial demonstration, the landscape of SERS-based *E. coli* detection has witnessed substantial technological growth and diversification [227]. Collectively, these advancements underscore the tremendous progress of this field, from simple nanoparticle suspensions to cutting-edge, multifunctional sensing platforms tailored for real-world applications.

Further integration of aptamers with emerging detection platforms for their signal enhancement, along with the identification and differentiation, is also under investigation. Several studies have integrated microchips, microfluidics, lateral flow devices, and other high-throughput platforms and reported unprecedented enhancements in Raman signals [215,228].

### 5.1. Microarrays

Microarrays are two-dimensional, compact platforms with multiple spots, each dedicated to a different sample and acts as an independent probe [5,229]. These spots can be functionalised with various aptamers, DNA oligonucleotides, antibodies, enzymes, and other molecular-recognition elements to advance this technology towards the parallel detection of multiple analytes simultaneously [5,103,230]. These static platforms provides robust optical interrogation of analytes without adding any complex fluidity to the system, making this technology simple, attractive, and easy to use [229]. In the case of SERS, microarrays are coupled with plasmonic nanostructures, and their spots serve as highly dense hotspots that enhance the SERS signals [190]. This further enhances the spectral resolution, detection sensitivity, and enhancement factor; moreover, it also addresses SERS limitations, such as lack of reproducibility due to batch variability, nonuniformity of hotspots, etc. A study in 2010 reported the in situ synthesis of a PDMS-AgNPs array with high cell adhesion capabilities for cell growth. This system, if directly employed under a Raman spectrophotometer, could enable rapid and straightforward detection of *E. coli* [231]. Nonetheless, the integration of these diverse techniques into the SERS framework has been a gradual and technically demanding process.

Recently, microarray-based detection technology has advanced further, with the integration of SERS-active components through techniques like inkjet printing, dip-pen nanolithography, and nanoimprint lithography. These fabrication techniques ensure high specificity, uniform interparticle spacing, and improved substrate biocompatibility [229].

Three-dimensional micro- and nanocavities serve even better, as they effectively confine the analyte in its compartments and reduce the chances of cross-contamination [187]. With the integration of SERS substrates, it achieves LODs as low as 1 CFU/mL and can detect a single *E. coli* cell. Soft-lithography-based PDMS microarray fabrication is a facile and tuneable approach that can easily produce uniformly distributed hotspots of various densities, and offers significant signal enhancement [152].

Moreover, as *E. coli* is a biological entity, the integration of a microarray with DNA-directed immobilisation (DDI) provides unprecedented enhancement and specificity. With the DNA capture elements, it works with molecular precision and enhances the hybridisation and signal transduction for more sensitive and specific *E. coli* detection. Aptamer-functionalised microarray systems are more common and proven prominent, particularly in the detection of virulent *E. coli* strains like O157:H7, with LODs in the picomolar range [183]. Wang et al. developed a microneedle array patch using a similar PDMS-based template moulding or soft lithography approach. This flexible patch can be used for impression-based sampling, bacterial extraction, and rapid detection with an LOD of 143 CFU/g [29].

Wen et al. developed a digital SERS-based inverted pyramid microcavity array system chip using a series of operations involving photolithography, etching, sputtering, nanoparticle deposition, etc., for *E. coli* detection. This study concluded that multilayer AuNPs picolitre cavities offer exceptional signal enhancement of up to 1.1 × 10^8^ and highlighted the potential of application of microarray-based SERS detection technology in bacterial detection from complex liquid systems [187]. Wang et al. developed an ultrasensitive, highly ordered 3-dimensional porous particle in a cavity array on a glove for the detection of sugar from sweat with the LOD ~5.7 ppt. This smart, nondestructive, and wearable technology can be directly used in bacterial detection from various surfaces with the highest accuracy and fewer chances of contamination [158].

Due to their effective detection and low LOD values, microarray-based SERS detection platforms are gaining mainstream attention for point-of-care (POC) diagnostics. Chen et al. developed a miniaturised gas membrane array system for the detection of *E. coli* and reported an LOD of 5–6 CFU/mL. This was one of the pioneering approaches in *E. coli* detection using flexible microarray systems and has the potential to be used alongside the filtration of liquid samples and can also contribute to public health monitoring [225].

Further advancement has been achieved with the integration of microfluidic interfaces for basic pretreatment, filtration, mixing, along with coupling with smartphone applications [183]. This extends the application of microarrays to portable, real-time surveillance of *E. coli* with high field deployability in resource-limited areas. Gu et al. used an integrated approach and developed a microarray-based microfluidic platform for multiplex detection. This study concluded that it has huge potential in clinical and biological diagnostics due to its high selectivity, uniform distribution of hotspots, excellent reproducibility, stability, and significantly lower limits, i.e., up to aM level concentrations [103].

**Figure 4:**
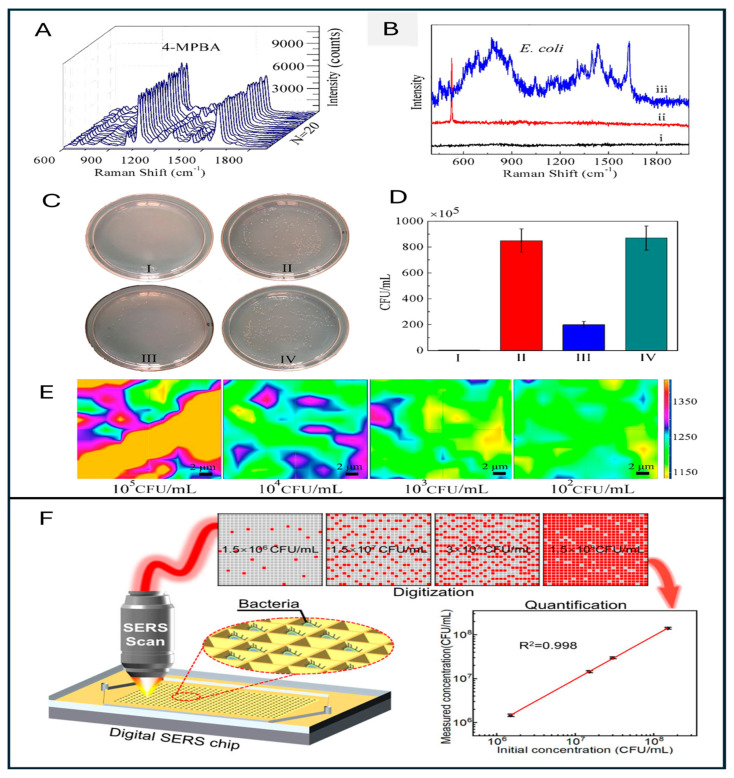
(**A**) Raman spectra of 4-mercaptophenylboronic acid on the Ta@Ag array surface, (**B**) Raman spectra of *E. coli* on the Ta@Ag array surface, (**C**) different films with bacteria inoculation for bioactivity evaluation, (**D**) quantitative analysis of bacterial bioactivity evaluated by using the plate count method, (**E**) raster-scanning Raman image of *E. coli* incubated on Ta@Ag array (adapted from [232] and reproduced with the permission of Copyright © 2020 American Chemical Society), (**F**) schematic diagram of AuNP-coated microarray-based SERS chip and *E. coli* mapping of different bacterial concentrations (adapted from [187] and reproduced with the permission of Copyright © 2024 American Chemical Society).

Figure 4 shows an *E. coli* detection study on Ta@Ag porous microarray and its impact on bioactivity. Additionally, in Figure 4F, shows a Au NP-coated microarray used for *E. coli* detection is shown.

A few pioneering studies have reported stable and durable array substrates for the dedicated detection of *E. coli* from food, blood, and water [233]. Abuhelwa et al. developed a novel fibre-optic-based microarray platform for SERS detection of *E. coli* and reported a very high sensitivity of 0.4–0.5 cells/mL. This 3D-printed device can detect various viral and bacterial species from a wide range of samples [211]. Another recent study developed a micro-fidget-spinner-based SERS platform for pathogen detection, identification, and quantification. It also reported the accurate detection and mapping of *E. coli* in urine samples [234].

### 5.2. Microchips

Microchips are the key component of portable platforms. They are central to the transformation of detection and biosensor technologies and represent miniaturised devices that are capable of handling small volumes of samples [235]. Integrating microchip technology SERS has redefined the paradigm of bacterial detection by enabling high portability and sensitivity.

These microchips are often considered biochips that offer a complete lab-on-chip platform by integrating complex pretreatment, sample processing, signal amplification, and detection onto a miniaturised platform. These are highly effective micro-platforms; however, their effectiveness and sensitivity depend on their fabrication technology [235]. Xue and Zhang reviewed the microchip-based SERS detection of *E. coli* and commented on its limitations, techniques, and future perspectives. This study concluded that using the advancements in the microchip fabrication process using 3D printing, lithography, etc., alongside the existing detection technologies like electrochemical sensing with SERS, will evolve this technology in order to be utilised as a prominent bacterial detection technique in complex matrices such as food, wastewater, blood, serum, urine, etc. [1]. These chips are often made up of glass, thermoplastics, paper, and PDMS [235].

These structures are coated or deposited with the plasmonic nanostructures to make them SERS-active. Filter paper-based nanoparticle-coated flexible microchips are the most basic version of this approach. Zhu et al. developed a ZnO@Ag functionalised paper-based microarray microchip for the SERS detection of pathogens such as *E. coli*, *S. aureus*, and *V. parahemolyticus*, and reported that, apart from the accurate detection, this platform is capable of photocatalytic sterilisation [236]. Mai et al. developed such a substrate for the in situ detection of dye and thiram from river water and apple skin up to a concentration of 10^−10^ M without using any specific aptamers or binders [237]. Using a similar approach, Ma et al. developed an advanced transparent and flexible AuNPs-PDMS-based chip and reported pesticide detection from food surfaces. These substrates can be directly utilised for pathogen detection, as they do not require analyte-specific aptamers [95].

Recent developments in microchip-based SERS platforms emphasise hybrid plasmonic architectures, like nanostar-decorated scaffolds, dendritic structures, and Au–Ag core–shell nanoparticles engineered within microchannels. These designs generate intense electromagnetic hotspots and leverage controlled flow dynamics to improve signal uniformity and reproducibility across the detection surface.

Microchips are highly effective for *E. coli* detection because of their seamless integration with other sophisticated technologies. Like microarrays, they can also be tailored with the applications of DNA probes, bacteriophages, immobilised antibodies, aptamers, etc., with the SERS active agents to ensure specific capture of analytes based on binding affinity. Due to this selective binding, detection within complex matrices such as urine, milk, blood, or wastewater without laborious sample processing or pretreatment. Notably, some researchers have developed designs accommodating the simultaneous enrichment and in situ detection on a single chip.

**Figure 5:**
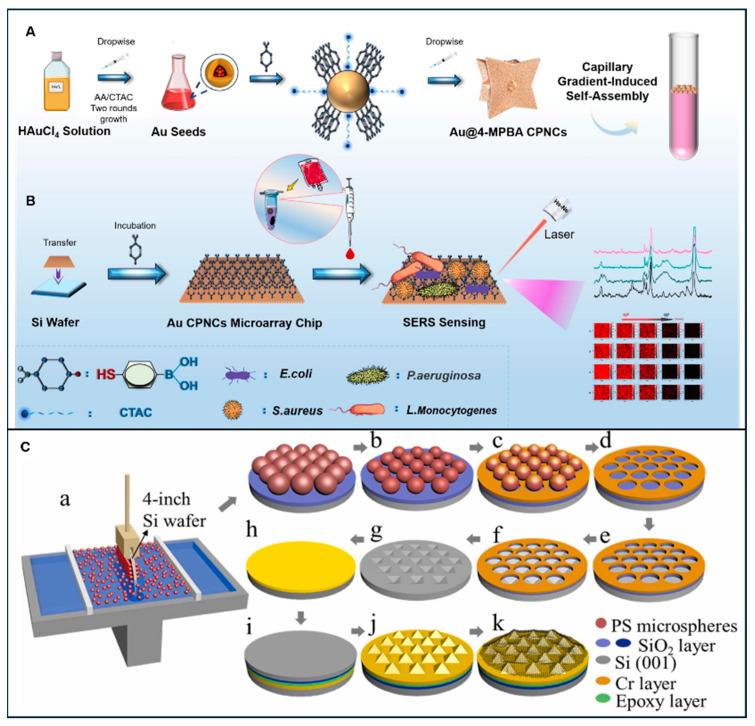
Schematic illustration of the synthesis, coating, and assembly of nanoparticles for a SERS microchip array dedicated to pathogen detection. (**A**) Capillary-gradient-induced-self-assembly-based Au CPNC fabrication process. (**B**) Preparation of Au CPNC microarray chips on silicon substrate films by self-assembly. Reproduced from [238] and reprinted with the permission of Copyright *© 2025 Elsevier*. (**C**) Schematic illustration of a hybrid SERS microchip fabrication process: (**a**) PS microsphere monolayer fabrication on a silicon wafer, (**b**) plasma etching, (**c**) electron-beam (e-beam) deposition, (**d**) removal of PS microspheres, (**e**) dry etching of SiO_2_ layers, (**f**) KOH etching, (**g**) dipping into HG for the removal of Cr and SiO_2_ layers, (**h**) Au film sputtering through a magnetron, (**i**) Au film and SiO_2_ film glued with epoxy, (**j**) Au film peels off, (**k**) graphene layer transfer on Au pyramids. Adapted from [239] and reproduced with the permission of Copyright *© 2021 Elsevier*.

Figure 5 illustrates representative studies on microchip fabrication and their application in SERS-based pathogen detection.

Zhang et al. developed novel face-centred gold convex polyhedral nanocrystals, assembled them on a silicon wafer microchip, and used them to detect different bacterial samples from whole blood. This study reported an enhancement factor of 5.38 × 10^7^ and LOD of 3 CFU/mL for *E. coli* with 100% accuracy [238]. Jiang et al. developed an integrated multifunctional microfluidic microchip for the SERS detection of pathogens. This study demonstrated magnetic separation, enrichment, derivatisation, and *E. coli* detection from blood samples on a single platform high accuracy [240].

Similar to the microarray-based SERS detection systems, recent studies on microchip-based SERS detection have also demonstrated excellent performance matrices with the limits of detection (LODs) as low as 1–10 CFU/mL and within the detection times ranging from a few minutes to under an hour. Yin et al. developed SERS microchips for detecting *E. coli* from milk and beverages using Ag foam substrates. This study employed a sandwich structure and concluded that sandwich structures on microchips are a facile and stable technique for the capturing, identification, and mapping of *E. coli* [241]. Jia et al. developed a flexible PDMS chip with the MXene@MOF@Ag ternary structure for using a two-step assembly process and detected *E. coli* up to 6.99 × 10^−8^ M [242]. Using a similar approach, Chen et al. developed a flexible PDMS chip with BP@CNT (black phosphorus @ carbon nanotubes) and detected *E. coli* up to 55.9 CFU/mL alongside the other urinary tract infection pathogens [243].

Due to their portability, they can easily be used with handheld spectrophotometers and can be integrated with smartphone-based, cable-based, or wireless data transmissions. However, despite their potential, there are a few challenges as well. Chip fouling, non-specific adsorption, nanoparticle degradation, and batch-to-batch reproducibility issues are among some of the major limitations. Furthermore, the long-term robustness of the system due to an extended period of detection, under varied environmental conditions, also needs to be considered before its widespread field deployment. With continued advances in microfabrication and nanomaterial engineering, microchip-based SERS detection is expected to emerge as an indispensable tool, with broad applications in border food safety, disaster-relief zones, remote clinics, and regions prone to pathogenic *E. coli* contamination.

### 5.3. Microfluidics

Microfluidics is a science of precision governed by the manipulation of fluid flow in microlitre- to nanolitre-scale volumes through meticulously fabricated channels thinner than a human hair. Microfluidic chips, also known as lab-on-chip platforms, integrate several functions on a single platform. This concept was first discovered by Eringen in 1964 [244]. These devices are made up of similar material to microchips, i.e., glass, thermoplastics, and PDMS, and act like miniaturised laboratories that accommodate complex biochemical operations on a chip [245]. Microfluidic devices are inherently highly efficient, and their integration with SERS amplifies molecular vibrational signatures through interactions with plasmonic microstructures. Together, microfluidic-enabled SERS provide a controlled, contamination-free microenvironment that can selectively capture, enrich, and spectrally interrogate the target analyte with high efficiency [246].

These platforms work on the principles of laminar flow, controlled mixing, and hotspot generation. These structures have a sample injection port and are sometimes connected to a peristaltic pump connected to a microchannel that guides the liquid analyte to flow across the SERS active regions, precoated or deposited with the plasmonic nanomaterials [139]. These hotspot areas work as focal zones for the spectral analysis, amplifying signal intensity and providing the robust spectral fingerprints of the analyte.

Signal intensity and enhancement in microfluidic SERS systems are strongly influenced by the design and composition of plasmonic nanostructures. Innovations such as Ag@Au core–shell particles, porous metal scaffolds, and aptamer- or DNA-linked nanoparticle clusters have been reported to yield enhancement factors up to 10^8^, while also improving signal reproducibility across the detection surface [244]. Concurrently, the functionalisation of nanoparticles with biorecognition elements—such as aptamers, antibodies, bacteriophages, and antimicrobial peptides—within chip-embedded microchannels enables the selective capture of *E. coli* from complex matrices like urine, milk, and wastewater [213]. Within these channels, the cells are subjected to the in-situ Raman analysis eliminating multiple pre-processing steps, such as centrifugation, washing and sample transfer. This single-step detection not only reduces the total detection time but also preserves the biochemical signature of *E. coli* as well [35].

Recent studies have reported in situ nanoparticle synthesis directly on microfluidic devices, further enhancing field deployability. Jayan et al. fabricated a microfluidic-SERS device incorporating in situ Ag nanoparticle synthesis guided by *E. coli* aptamers and reported an LOD value of 1.1 CFU/mL [215]. Wang et al. similarly demonstrated in situ synthesis of Au NPs, Au nanorods, and Ag@Au NPs within 10 min, yielding morphologically uniform and monodisperse nanoparticles. This study reported an enhancement factor of 10^6^ and significant amplification of characteristic *E. coli* Raman peaks at 520 cm^−1^, 1171 cm^−1^, 1247 cm^−1^, 1397 cm^−1^, 1619 cm^−1^, etc. [74].

Microfluidic-based SERS platform fabrication originated from soft lithography has since evolved through advanced techniques such as nanoimprint lithography, laser micromachining, and 3D printing [247]. These techniques enable mass-production of robust, multilayer chips with integrated functions-including pretreatment, lysis, enrichment, and detection-forming a one-spot microfactory [103]. Recent studies have optimised bacterial residence time and analyte–nanoparticle interactions using serpentine mixers, herringbone patterns, and droplet reactors. Furthermore, the development of paper-based microfluidic and textile integrated platforms is backing a new era of ultra-portable, disposable diagnostics for low-resource settings [247,248]. Additional advances include the integration of magnetophoresis or dielectrophoresis, using an electric field to guide the flow and trap *E. coli* cells precisely in the hotspot-dense region [74].

**Figure 6:**
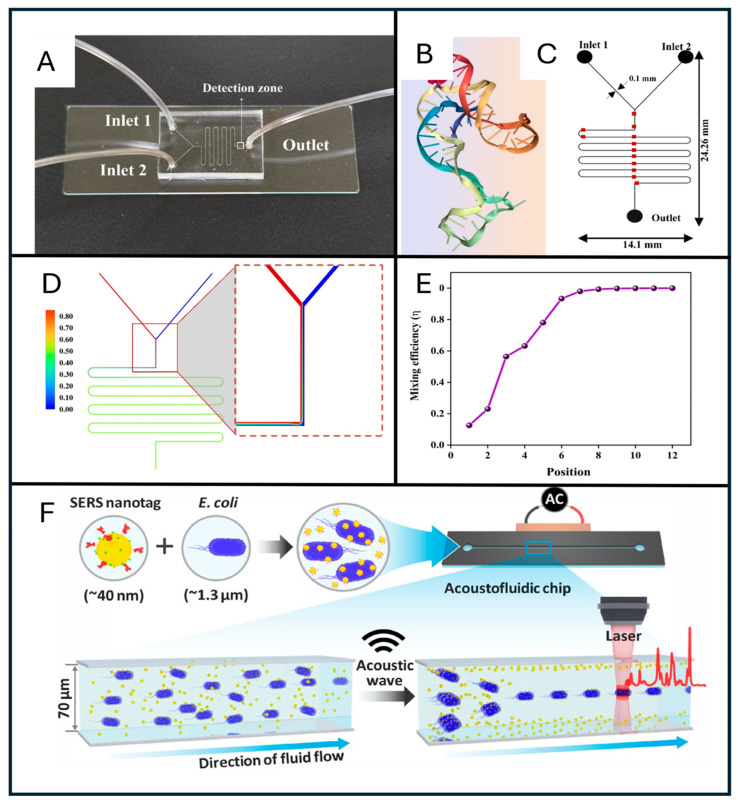
(**A**) Microfluidic device fabricated for in situ Ag NP synthesis, (**B**) secondary structure of the aptamer required for *E. coli* detection, (**C**) two-dimensional (2D) geometric view of the microfluidic channel with data collection points, (**D**) mass fraction of the precursor solution, (**E**) mixing efficiency at the marked position of the microfluidic channel (adapted from [215] and reproduced with the permission of Copyright *© 2025 Elsevier Ltd.*), (**F**) recent integrated study on SERS detection utilising the acoustofluidic behaviour of *E. coli* with SERS tags and wave-on and wave-off conditions (adapted from [251] and reproduced with the permission of Copyright © 2025 American Chemical Society).

Microarray-assisted microfluidic devices further enhance SERS performance. Several recent SERS studies have integrated microarray and microfluidic approaches. Dong et al. developed a microsphere-array lens integrated with microfluidics for *E. coli* detection and demonstrated proof-of-principle experiments using SERS and magnetic tags. With excellent signal reproducibility, position tolerance, and LOD value of 5 cells/mL, this study concluded that the device is suitable for point-of-care pathogen detection [249]. Although microfluidic-based SERS is already highly accurate, it continues to evolve through integration with complementary technologies such as microarrays, flow cytometry, CRISPR assays, and electrokinetic control. Fong et al. integrated flow cytometry with the microfluidic-based SERS and detected highly dense *Salmonella choleraesuis* up to 2000 CFU/mL with high accuracy (R^2^ value 99%). They developed a spectral flow cytometric chip incorporating nanoaggregate-embedded beads for flowing Raman dye-tagged analytes [250]. Owing to its high specificity and accuracy, this approach can be extended to other bacterial classes. Figure 6 illustrates representative studies on microfluidic device fabrication, simulation, and integrated approaches linking acoustic wave manipulation with SERS signal enhancement.

Dogan et al. fabricated a capillary-driven microfluidic device incorporating magnetic SERS tags and reported *E. coli* detection up to 10^1^–10^7^ CFU/mL within 60 min [25]. Zhuang et al. integrated the CRISPR/CAS assay with microfluidics and SERS to develop a paper-based, flexible, point-of-care device for pathogen detection, achieving an LOD of 3–4 CFU/mL in spiked milk and meat samples [248].

A recent study by Park et al. developed a novel Acoustofluidic microfluidic-based SERS device and reported an LOD of 1.75 × 10^5^ for *E. coli* detection. SERS nanotags and *E. coli* were passed through a microfluidic channel, and a piezoelectric transducer was used to generate acoustic waves that align larger *E. coli*–SERS tag aggregates, thereby making the detection process more efficient and reliable (Figure 6F) [251]. This pioneering study in the SERS domain is expected to open new opportunities for integrated acoustofluidic–SERS diagnostics.

Despite several advantages, microfluidic-based platforms, similar to microarrays and microchips, also face challenges such as non-specific adsorption, plasmonic degradation, and channel fouling. However, with continuous advancements in durable nanoparticle synthesis, surface chemistry, material stability, and fluid dynamics, this process is steadily crossing most of these hurdles.

### 5.4. EC-SERS

Electrochemical-surface-enhanced Raman spectroscopy (EC-SERS) is an advanced version of SERS that applies controlled electric current and potential during Raman analysis. EC-SERS leverages the synergistic effects of localised surface plasmon resonance (LSPR), while controlling the electron transfer processes at the electrode interface. This integrated approach enhances the Raman signals and provides valuable insights into the redox behaviour of bacterial cells. Consequently, EC-SERS has proven effective in addressing key challenges in bacterial diagnostics, including sensitivity, selectivity, reproducibility, and detection time [252,253].

EC-SERS involves the application of an electric potential to a SERS-active electrode, which modulates the electronic environment at the metal–analyte interface. Fermi-level tuning of metal substrates-particularly gold and silver plasmonic nanoparticles, enhances molecule–substrate charge-transfer efficiency, leading to increased signal intensity. In the case of *E. coli*, it translates the spectral insights into clearer vibrational characteristics associated with its components, such as membrane proteins, nucleic acids, flavins, and lipopolysaccharides [254].

Several recent EC-SERS studies demonstrated that the application of a small negative potential between −0.3 V and −0.8 V can significantly amplify the SERS signals of flavin adenine dinucleotide (FAD), a redox-active metabolite associated with *E. coli* metabolism. This finding has paved the way for probing functional biochemical states of analyte molecules rather than relying on static molecular fingerprints. Such electrochemical characteristics enable differentiation between live and dead bacterial cells and their responses to environmental stress [255].

Recent advancements in EC-SERS include the extensive use of screen-printed electrodes (SPEs), flexible conducting polymers such as plasmonic nanoparticle-coated PEDOT:PSS, indium tin oxide (ITO) glass. The selection of suitable nanomaterials, along with their morphology and dense packing, critically determines hotspot density, enhancing signal intensities from 10^6^ to over 10^9^ in optimised systems [256]. One major advancements in EC-SERS is the incorporation of three-dimensional plasmonic nanostructures, which provide high surface area conducting networks, enabling *E. coli* detection less than 7 CFU/mL in under 30 min [257].

Furthermore, the integration of EC-SERS with microfluidic platforms enabling automated sampling, real-time analysis, and point-of-care detection has significantly advanced the field. Such integration reduces contamination risks, loading errors and sample preparation variability, while enabling continuous monitoring. These microfluidic-based EC-SERS hybrid devices have demonstrated promising results for detecting *E. coli* from complex matrices [258]. Selectivity can be further enhanced by functionalising electrodes with biorecognition elements such as aptamers, DNA oligonucleotides, and antibodies, resulting in unprecedented signal enhancement through selective targeting of *E. coli* antigens. These modifications not only improve selective binding but also facilitate investigation of electrochemical effects on charge-transfer mechanisms responsible for SERS enhancement. Consequently, EC-SERS enables analysis of redox-sensitive metabolic shifts, providing mechanistic insights that are not accessible through conventional SERS.

**Figure 7:**
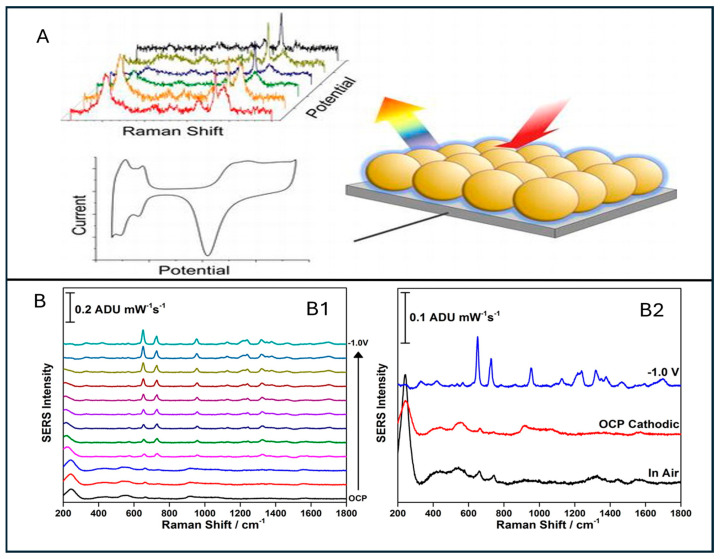
(**A**) Schematic of the integrated EC-SERS system and its advantages; (**A**) is adopted from [259] © Crown Copyright 2017 and reproduced with the permission of the Controller of Her Majesty’s Stationery Office. Published by Informa UK Limited, trading as Taylor & Francis Group. (**B**) The results of EC-SERS measurements of *E. coli*, (**B1**) the EC-SERS signal of *E. coli* K-12 on Ag@SPE with PBS, (**B2**) comparison of EC-SERS spectra in air, PBS, and OCP. Adapted from [260] and reproduced with the permission of Copyright © 2018 American Chemical Society.

Although EC-SERS is still evolving, one of its most transformative evolutions is the integration of microchip-SERS platforms with electrochemical control. This approach combines tuneable potential, laser excitation, and lithography-defined microstructures, resulting in enhanced signal intensity and redox-sensitive monitoring of bacterial analytes. Figure 7 illustrates a representative EC-SERS integrated setup and the corresponding enhanced Raman signals of *E. coli*.

In EC-SERS, printed flexible nanomaterial-based devices are integrated and powered by potentiostats and handheld Raman spectrometers. This makes it a highly promising detection technique offering high sensitivity, portability, and cost-effectiveness in terms of device fabrication and its suitability as a point-of-care diagnostic tool [261]. Apart from the traditional integrated electrochemical Raman setup, many other innovative integrations are also under exploration for various detection applications. Beyond signal enhancement, integrating EC-SERS with platforms such as microarrays, microfluidics, photoluminescence, and liquid chromatography can improve signal-to-noise ratios and enable more sensitive detection, establishing it as an advanced and emerging realm of SERS detection [262,263].

Despite having considerable potential, EC-SERS still has a few limitations. Under an applied potential, the formation of electrochemical byproducts and the stability of plasmonic coatings can be affected, which need to be controlled and monitored. Further, under the applied potential, the biological molecules can be degraded. However, ongoing electrode-based research on self-healing electrodes, stable plasmonic materials, and real-time feedback control systems suggests these issues can be effectively resolved.

### 5.5. Lateral Flow Devices

Lateral flow assay (LFA) devices are paper-based, flexible devices that are being used for the detection of analytes in urine, blood, etc. This technique was first reported in the 1960s for protein detection in serum [264]. With the evolving landscape of point-of-care diagnostics, its fusion with SERS is transforming the paradigm of rapid, facile, and affordable spectral precision of *E. coli*. Historically, LFAs are best known for their role in pregnancy tests and other basic tests for infectious diseases [265]. However, they are limited due to low sensitivity and restricted outputs. Their integration with SERS has reinvigorated their analytical performance by enabling quantitative output, higher sensitivities, lower cost, and a portable format [266]. Depending upon the requirements and resources, a wide range of lateral flow assays in the form of dipsticks, biosensors, immunoassays, immunodiffusion strips, and immunochromatographic techniques have been developed [264,265]. Conventional colorimetric signal-based lateral flow assays are qualitative or semi-quantitative. However, their integration with new signal models, such as Raman, has enhanced their accuracy and sensitivity to a great extent [267].

LFA-based SERS technology has attracted the attention of researchers as a promising detection technique because it utilises the highly specific plasmonic nanostructures that are typically functionalised with the Raman active molecules, such as aptamers, antibodies, phage-derived peptides, etc., providing specificity alongside its potential for multiplex detection [268]. These hybrid nanoprobes are engineered to bind specifically with the analyte molecules or their surface antigens, forming a sandwich complex that migrates along with the nitrocellulose membrane on the LFA strip. After reaching the test line, these sandwich molecules are immobilised by the capture molecules and subjected to the Raman analysis. On one LFA strip, many test lines specific to the analyte can be present and can capture the specific analyte, depending on the affinity of the capturing molecules [269]. These strips are highly recommended for the molecular fingerprinting of *E. coli* and its other strains. With the innovations in the synthesis of plasmonic nanoparticles, the utilisation of core-shell nanoparticles can maintain the higher stability and bio-functionality and enhanced electromagnetic field hotspots. To generate a densely populated and uniformly distributed hotspot area, various types of anisotropic plasmonic nanostructures are being introduced on these strips [270]. Chen et al. developed a lateral flow assay incorporating the immobilised antimicrobial peptide cecropin1 on Fe_3_O_4_@Au nanoparticles to selectively capture *E. coli*, achieving a limit of detection of 16 CFU/mL. This performance was aided by magnetic Fe_3_O_4_@Au–based enrichment, alongside enhanced antibacterial activity, good reproducibility, and high analytical sensitivity [270].

**Figure 8:**
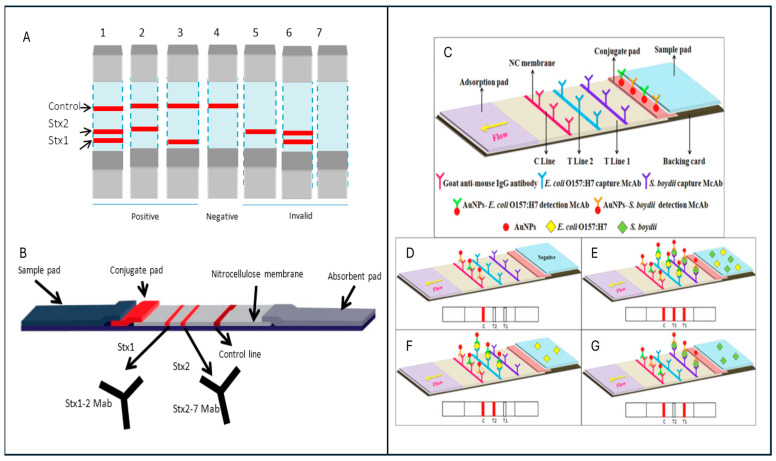
Schematic representation of lateral flow devices. (**A**) Expected results depend upon the coating. (**B**) The LFIA device with all its components. Adapted from [273] and reproduced with Permission from MDPI. (**C**) The LFIA device with all of its components, flow, and major coating material used for *E. coli* detection. (**D**–**G**) Typical results of multicomponent analysis by LFA. Adapted from [274] and reproduced with permission of *Copyright © 2015 Elsevier Ltd*.

Technical innovations in biomolecular detection and identification are making the functionalisation strategies for the selective capturing of *E. coli* more sophisticated. For instance, the utilisation of dual-aptamer systems and advancements in the chemistry of linker molecules enable enhanced affinity, orientation, and density of bioreceptors on the nanoparticle surfaces, enhanced target capture efficiency, and reduced false positives [271,272]. Wang et al. developed a GO@Au/Ag-based bi-channel LFA for the multiplex detection of four bacterial species—*E. coli*, *S. aureus*, *L. mono*, and *S. typhi*, respectively—and reported an LOD of 9 cells/mL in 20 min. Simultaneous utilisation of two different Raman reporter molecules not only enhances the Raman signals but also establishes a simplified multiplex detection point-of-care technique [267]. Figure 8 shows the LFA strips, their structure, and components.

Li et al. reported the synthesis of novel Au super-particles (i.e., small AuNPs encapsulated in polymer beads) and their application in LFA-based SERS detection of *E. coli* in milk. The study reported a remarkable enhancement in absorbance values and demonstrated high accuracy and specificity with the application of modified plasmonic nanostructures [269]. As mentioned earlier, LFA-based SERS enables multiplexed detection on a single strip, which is the most compelling development in the LFA-based SERS detection. By tailoring the unique Raman tags for different pathogens, including different *E. coli* strains, multiple targets can simultaneously be identified on a single strip. Shen et al. designed 3D-membrane-like SERS tags and used them on LFA for *E. coli* detection, reporting a remarkably low LOD value of 30–40 CFU/mL. This study demonstrated effective and quantitative multiplex detection of *E. coli*, *S. typhi*, and *P. aeruginosa* on this point-of-care LFA strip [268]. Several researchers have begun developing LFA for multiplex detection by making multiple test lines on the strip [275]. Further complex spectral data can be deconvoluted using machine learning algorithms and other multivariate chemometric analyses such as principal component analysis (PCA), partial least squares regression discriminant analysis (PLS-DA), etc. [276]. Apart from PCA and PLS-DA, hierarchical cluster analysis (HCA) and differential functional analysis (DFA) can also be used for detecting complex samples, such as food, blood, urine, and serum [277].

Considering the fabrication perspective of LFA strips, advancements in laser patterning, membrane synthesis, and inkjet deposition enable large-scale, reproducible production with SERS tags. These techniques not only produce the precisely aligned nanoparticle conjugates but also allow the flow kinetics to be tailored to ensure the reproducibility and sensitivity of the analysis.

Despite these promising elements, several challenges remain. Key issues include the reproducibility of SERS signals across batches, spectral interferences from the sample matrix, and uniform immobilisation of biomarkers or bioreceptors on the substrate. Furthermore, because of ambient environmental conditions, the long-term stability of nanoprobes is often questioned; therefore, shelf-life extension and commercial viability are still under investigation. However, integrating LFAs with the microarray SERS systems can enhance the signal reproducibility, while the utilisation of stable plasmonic nanostructures addresses many concerns of commercial viability.

## 6. Detection Cost, Process Robustness, and Regulatory Acceptance

Recent efforts have produced low-cost and more robust SERS substrates, such as scalable electrochemical fabrication kits that reduce per-test consumable cost to fractions of a cent, without major compromises in signal reproducibility [278]. Apart from this, another emerging need is durability in real-world and on-site scenarios. Developing a stable, robust, and low-cost technique is already underway in various research facilities. However, a regulatory guideline is still a steep climb to achieve [19,278]. SERS-based detection not only requires high sensitivity but also long-term stability, easy spectral identification, differentiation, and batch-to-batch substrate reproducibility. This needs to be validated with various real-world biological analytes and commercially available substrates to establish a global standard [19]. Furthermore, while the miniaturised Raman detection platforms offer a potential alternative and represent the future of Raman-based point-of-care detection, they still show limitations in terms of optical resolution and the impact of environmental conditions on the signal sensitivity [279]. The real-world and on-site deployability of Raman-based detection requires a standardised protocol with improved chemistry, control over nanofabrication, and regulatory frameworks [19,278,279]. These are the major regulatory challenges that need to be discussed and addressed before the transition of SERS biosensors from laboratories to the real world as a reliable detection tool for environmental monitoring.

## 7. Comparison, Challenges, Future Aspects, and Commercial Developments in SERS-Based *E. coli* Detection

A wide range of SERS platforms has been applied to E. coli detection, each offering distinct advantages and limitations. Microfluidic devices, microarrays, and chip-based systems provide the highest analytical sensitivity, enabling exceptionally low limits of detection (LOD) with excellent spectral precision [215]. However, these systems also impose significant environmental and operational requirements, such as controlled flow conditions. EC SERS yields some of the most robust analytical performance but requires specialised cell assemblies and a potentiostat for measurement, adding complexity to deployment [35].

Nanoparticle-tagged LFAs are rapid, easy to use, and well-suited for field applications, though their sensitivity and LOD values remain moderate. Integrated Raman systems combining electrochemistry or microfluidics aim to merge portability with high sensitivity, but their performance is still strongly dependent on optical stability and substrate quality [276]. Taken together, these comparisons reveal a recurring pattern: platforms that deliver high sensitivity typically demand tight fabrication control and precise operating conditions, whereas systems optimised for usability often sacrifice detection limits. A realistic future solution will need to balance these trade-offs rather than pursuing a single optimisation target.

Despite steady progress, several persistent challenges continue to limit translation. SERS substrates often show batch-to-batch variability, long-term signal stability can be inconsistent, and many systems perform poorly outside of controlled laboratory environments [280]. Regulatory validation remains another significant bottleneck for food and clinical applications, where analytical robustness must be demonstrated across large and diverse sample sets. Even so, the field is strengthening its foundations: more consistent fabrication protocols, improved surface chemistries, and cleaner device architectures are helping push these technologies closer to practical deployment [35]. The trajectory is promising, but the technical demands remain high, and only platforms capable of meeting these stringent requirements are likely to achieve reliable real-world use.

Many of the advanced techniques described above share common underlying features. Nearly all platforms demonstrate a convergent trend toward miniaturisation, portability, and reduced sample volumes, reflecting a systematic transition from laboratory-based benchtop systems to compact, field-deployable architectures suitable for point-of-care (POC) diagnostics [25]. Additionally, the integration of these platforms with microfluidic systems provides automated fluid handling, enabling continuous sample flow, in situ pretreatment (filtration, washing, mixing), real-time monitoring, and minimised contamination risks, thereby eliminating manual handling errors and accelerating time-to-result [215]. Many systems also incorporate smartphone-based readout and data analysis capabilities, extending their utility to resource-limited settings where conventional laboratory infrastructure is unavailable. Advances in microfabrication and nanofabrication techniques, such as photolithography and inkjet printing, facilitate reproducible, large-scale development of SERS substrates with uniform hotspot distributions [35,280]. Furthermore, the application of multivariate chemometric analyses, including principal component analysis (PCA), partial least squares (PLS) regression, hierarchical cluster analysis (HCA), and machine learning algorithms, is increasingly employed across platforms to deconvolute complex spectral data, improve classification accuracy, and enable multiplex detection of multiple pathogens or *E. coli* strains on a single device [276,277].

Commercial development of advanced *E. coli* detection tools is beginning to take clearer shape, although progress differs across platforms. Several components of the workflow are now well aligned with industry requirements. Plasmonic substrates can be fabricated with improved uniformity, microfluidic chips can be moulded at scale, and compact Raman units have reduced earlier hardware limitations [35]. These advances have enabled several groups and companies to test early prototypes for food, water, and clinical screening without sacrificing spectral quality [266].

However, full commercial maturity still requires more consistent performance. Reproducible substrates, extended signal stability, regulatory approval, and dependable field operation remain the key challenges [35,280]. Recent improvements in surface coatings, more robust fabrication protocols, automated spectral processing, and sealed cartridge-based sampling are gradually closing these gaps [215]. The progress is steady and practical: platforms are now moving beyond single-use academic demonstrations toward systems that can be manufactured, certified, and reliably deployed outside of the laboratory.

## 8. Conclusions

Pathogen detection techniques each come with distinct advantages and limitations. Among them, Surface-Enhanced Raman Spectroscopy (SERS) stands out as a highly promising method, offering unparalleled specificity through the molecular fingerprints of bacteria and other analytes. This paper provides a comprehensive overview of the advancements in *E. coli* detection using SERS, highlighting the evolution of this technique and its transformative potential in biosensing.

The synergistic integration of substrate fabrication, sample preparation, laser optimisation, and data analysis has significantly enhanced the reproducibility, sensitivity, and practical applicability of SERS. Starting from colloidal nanoparticle-based approaches to recent innovations such as microarrays, microchips, microfluidic devices, electrochemical-SERS (EC-SERS), and lateral flow assays, this review has explored their fabrication strategies, limitations, and potential for field deployment. Furthermore, integrated approaches, such as microarray-based EC-SERS platforms, offer a pathway to overcome many existing challenges.

By combining these advanced strategies, many limitations in current SERS-based *E. coli* detection methods can be addressed. Such integration will pave the way for the development of miniaturised, portable SERS platforms capable of transitioning from laboratory settings to scalable, point-of-care applications. These innovations hold the potential to revolutionise bacterial detection and broaden the applicability of SERS in real-world diagnostics.

## Figures and Tables

**Table 1:** Different metallic nanoparticle incorporation techniques used for SERS substrate development.

S.N.	Substrate Type	Method	Process	Sensitivity	Uniformity	Reproducibility
**1**	Colloidal nanoparticles	Colloidal Nanoparticles	Easy NP synthesis is required	Low	Poor	Poor
		Microstructures of plasmonic materials (e.g., copper-, silver-, or gold-based lithography)	Sophisticated and hard Lithography, sputtering, hot-press, etc.	Very high	Very high	Very high
		Nanoparticle coating on microstructure surfaces	Complex Nanostructured substrate synthesis (ablation, lithography) and NP deposition are time-consuming	Moderate to high	Moderate to high	High
**2**	Nanoparticles on porous materials	Nanoparticle coating on porous material	Complex Fabrication is time-consuming	Moderate to high	Moderate to high	Moderate
		Nanoparticle coating on commercially available substrates	Easy NPs’ attachment to the substrate is required	Moderate to acceptable	Poor to moderate	Moderate to high
**3**	Nanoparticles on flexible substrates	Mixing and drop-casting or filtering through/on flexible surfaces (e.g., filter paper or membrane-assisted SERS)	Easy NP synthesis is required	Moderate to high	Poor	Poor
		Nanoparticle coating on flexible surfaces	Easy NP synthesis is required, followed by coating	Moderate	Poor to moderate	Moderate
		Nanoparticle coating on microstructures’ flexible surfaces	Complex Nanoparticle synthesis, microstructure making (lithography, template moulding, electrochemical deposition, etc.), followed by nanoparticle coating (if required)	High	Moderate to high	High
		Nanoparticle coating on flexible electrodes (EC-SERS)	Easy NPs synthesis is required	Very high	High	Very high

**Table 2:** Peak assignment table with all the reported peaks in recent literature.

S.N.	Bands	Assignment/Description	References
1	653	Xanthine (DNA/RNA component)	[176,177]
2	655	Hypoxanthine, xanthine, guanine, tyrosine	[137,178,179,180]
3	730	Ring vibration of adenine Adenine, hypoxanthine, adenine monophosphate Adenine, hypoxanthine, adenine monophosphate A ring breathing (nucleic acid) Ring breathing of adenine (nucleic acid)	[59,177,181,182,183,184,185]
4	733		[59,185,186,187,188]
5	734		[185,189,190,191,192]
6	735		[137,146,171,185,191,193,194]
7	736		[146,185,195,196]
8	738		[146,197,198,199,200]
9	768	Pyrimidine ring breathing mode	[140,201]
10	796	Tyrosine C–O–P–O–C—RNA binding	[108,178,202]
11	900	Polysaccharide (bacterial cell wall)	[137,140,203]
12	955	Xanthine Xanthine; protein skeleton C-single bond C-stretch, N–C-α-C stretch α-helix	[59,176,181]
13	960	Xanthine; protein skeleton C-single bond C-stretch, N–C-α-C stretch α-helix/δ(C = C)	[29,59,184,191,204]
14	1000	Protein/ring vibration of phenylalanine	[176,182,203,205]
15	1004	ns(C-C), symmetric ring breathing (phenylalanine of collagen)	[11,13,186,197]
16	1010	Phenylalanine (proteins)	[140,194,197]
17	1025	Carbohydrate’s peak	[29,185]
18	1031	d(C-H), phenylalanine (protein assignment) C-H in-plane bending mode of phenylalanine Carbohydrate residues of collagen Phenylalanine, C-N stretching of proteins C-H in-plane bending mode of phenylalanine	[11,178,202]
19	1146	Carbohydrates peak for solids	[187,206]
20	1240	Amide III/amide III (random coil)	[137,146,179,186,193,207]
21	1248	Amide III (of collagen)	[108,146,184,200]
22	1280	C–N and N–H stretching (amide III); CH_2_ and CH_3_—protein deformation; guanine breathing ring	[108,208,209]
23	1300	-(CH_2_)n- in-plane twist vibration (lipid band) d(CH_2_)-lipids, fatty acids CH_2_ twisting modes d(CH), t(CH_2_)(a-helix) CH_2_ twisting (lipids) CH_2_ twisting and wagging (lipids)	[11,183,201,207]
24	1310	CH3/CH2 twisting, wagging and/or bending mode of collagens and lipids	[138,210]
25	1320	Adenine, xanthine, adenine monophosphate (C–N) stretch	[59,184,186,197,211]
26	1326	Ring vibration of adenine Xanthine, adenine, AMP	[182,203,212]
27	1330	Typical phospholipid region associated with DNA and phospholipid collagen (nucleic acids and phosphates)	[11,146,177,181,200]
28	1335	Adenine	[137,146,185,193]
29	1395	C55O symmetric stretch/CH_2_ deformation	[149,211]
30	1415	C55O symmetric stretch quinoid ring	[186,197]
31	1450	CH_2_ scissoring vibration (lipid band)	[146,178,184,187,189,209]
32	1466	CH_2_ deformation of lipids, proteins, carbohydrates	[182,203,213]
33	1504	–C–C conjugated stretching—carotenoids	[108,214,215]
34	1570	C–C stretching	[186,197,198]

**Table 3:** Compilation of some recent studies on SERS detection of *E. coli*.

S.N.	SERS Substrate Fabrication Techniques	Substrate	Substrate Synthesis	Laser	Detection Time	*E. coli* Source	Sample Preparation	Major Bands Observed	LOD	E.F.	Reference
**1**	Manual mixing and drop-casting	AgNPs	Chemical reduction method	785 nm	60 min	Bacterial culture	*E. coli* supernatant mixed with AgNPs and drop-casted	1013 cm^−1^, 1025 cm^−1^, 1173 cm^−1^, 1335 cm^−1^, 1558 cm^−1^, 1624 cm^−1^	-	100%	[185]
**2**	Mixing and drop-casting	AgNPs	Chemical reduction method	532 nm	1 s	Bacterial culture	Resuspended the washed *E. coli* in mile-Q water, mixed with AgNPs, and drop-casted	735 cm^−1^, 800 cm^−1^, 1335 cm^−1^, 1455 cm^−1^, 1633 cm^−1^	-	-	[193]
**3**	Nanoparticle synthesis on analyte surface	AgNPs	In situ synthesis	532 nm	5 s	Chicken carcass wash-water	Washed *E. coli* cells were coated with AgNPs using an in situ process and drop-casted	1153 cm^−1^, 1256 cm^−1^, 1345 cm^−1^, 1535 cm^−1^, 2930 cm^−1^	-	-	[180]
**4**	Mixing and drop-casting	AgNPs	Chemical reduction method	785 nm	40 s	Bacterial culture	*E. coli* cells were washed and mixed with AgNPs and drop-casted	658 cm^−1^, 734 cm^−1^, 958 cm^−1^, 1332 cm^−1^, 1450 cm^−1^	-	-	[189]
**5**	Mixing and drop-casting	AuNPs	Chemical reduction method	780 nm	1 s	Ground beef	*E. coli* was mixed and incubated with AuNPs and drop-casted	738 cm^−1^, 1004 cm^−1^, 1010 cm^−1^, 1320 cm^−1^, 1415 cm^−1^, 1570 cm^−1^,	-	-	[197]
**6**	Drop-casting and manual mixing	Ag-Au microcapsules (layer-by-layer assembly)	Layer-by-layer assembly	785 nm	5 s	Bacterial culture	Washed and thawed *E. coli* cells were drop-casted on microcapsules	1200 cm^−1^, 1248 cm^−1^, 1280 cm^−1^, 1355 cm^−1^, 1396 cm^−1^, 1435 cm^−1^, 1654 cm^−1^	-	10^9^ EF	[108]
**7**	Mixing and drop-casting	AuNP-coated filter paper	Self-assembly	785 nm	--	Bacterial culture	Washed *E. coli* cells were resuspended and mixed with AuNPs, incubated, and drop-casted	733 cm^−1^, 1004 cm^−1^, 1114 cm^−1^, 1240 cm^−1^, 1320 cm^−1^, 1415 cm^−1^, 1570 cm^−1^		-	[186]
**8**	Flat gel surface coated with NPs (aptamer-mediated specificity)	Apt-AgNPs-CS gel	Mixing, coating, and drop-casting	633 nm	120 min	Milk and orange juice	Washed *E. coli* cells were mixed with plasmonic complex and incubated in the dark, washed and mixed with aptamers and incubated in the dark, and washed with PBS; the sandwich structure was analysed	738 cm^−1^, 1100 cm^−1^, 1570 cm^−1^	3.46 CFU/mL	-	[198]
**9**	Mixing and drop-casting	Au@Ag-stuffed Nanopancakes	Mixing and self-assembly	532 nm	--	Bacterial culture	*E. coli* cells were mixed with SERS tags, incubated, and washed. Mixed with Au@Ag nanopancakes and drop-casted	415 cm^−1^, 687 cm^−1^, 994 cm^−1^, 1017 cm^−1^, 1067 cm^−1^, 1188 cm^−1^, 1217 cm^−1^, 1594 cm^−1^	7 CFU/mL	-	[106]
**10**	Nanostructured plasmonic meta-surface	Au + metasurface cavities	Electron beam lithography	785 nm	10 s	*E. coli*	*E. coli* toxins were drop-casted on nanostructures, 2-nanostructure prefunctionalisation, and *E. coli* toxin drop-casting	768 cm^−1^, 1022 cm^−1^, 1053 cm^−1^, 1115 cm^−1^, 1300 cm^−1^, 1392 cm^−1^	1.4 nM	9 × 10^7^	[201]
**11**	Nanoparticles on a flexible surface (filter paper)	Au@Ag NPs + filter paper	Seed growth method followed by dip-coating	785 nm	--	Bacterial culture	*E. coli* cells were drop-casted on NP dip-coated filter paper	655 cm^−1^, 735 cm^−1^, 900 cm^−1^, 1240 cm^−1^, 1335 cm^−1^	10^4^ CFU/mL	-	[137]
**12**	Nanoparticle coating on flexible surface (antibody-mediated specificity)	GO@Au nanosheet with antibodies	Two-step seed growth method followed by antibody mixing 2-antibody-sprayed strip loading	-	30 min	Bacterial culture	Antibodies were mixed with the GO@Au nanosheets and *E. coli* cells and drop-casted 2-antibody-sprayed strip was loaded with a mixture of *E. coli* cells and GO@Au nanosheet-coated strip	1079 cm^−1^, 1310 cm^−1^	10 cells/mL	7.07 × 10^7^	[138]
**13**	Mixing and drop-casting	Anup’s with Thioglucose, polyvinylpyrrolidone, and citrate	Chemical reduction	808 nm	10 min (60 s × 10 cycles)	Bacterial culture	*E. coli* cells were mixed with AuNPs, drop-casted, and heat-fixed	500 cm^−1^, 600 cm^−1^, 768 cm^−1^, 900 cm^−1^, 1010 cm^−1^	-	-	[140]
**14**	Drop-casting and manual mixing	Ag^+^ surrounding single poly(4-cyanostyrene) NPs	Emulsion polymerisation	532 nm	5 s	Drinking water, sour milk, and lake water	Washed *E. coli* cells were mixed with Ag+, incubated, and centrifuged. Supernatant was further blended with poly (4CSN) NPs and filled in capillary	-	100 cells	-	[218]
**15**	Mixing and drop-casting (antibody-mediated specificity)	AuNPs+antibiotics	Mixing	785 nm	1 s	Bacterial culture	Washed *E. coli* cells were resuspended, mixed with AuNPs, and drop-casted	738 cm^−1^	-	-	[199]
**16**	Drop-casting and manual mixing	AuNPs/PIB−POE−PIB/NMPs	Multistep assembly of nanoparticles using hydrophilic and hydrophobic polymer dispersants	633 nm	--	Bacterial culture	*E. coli*	736 cm^−1^	10^−8^ M	8.9 × 10^6^	[195]
**17**	Nanoparticles on microstructured surface (capillary-driven detection)	PDMS-based microfluidic channel and AuNPs	Chemical reduction method and micro/soft lithography	785 nm	60 min	Bacterial culture	Fixed *E. coli* O157:H7 cells were washed, centrifuged, and mixed with SERS active probes. Mixture was incubated, centrifuged, and pelleted down	-	0.5 CFU/mL	-	[139]
**18**	Mixing and drop-casting	PVP-modified AgNPs colloid	Chemical reduction followed by surface modification/functionalisation	-	--	Bacterial culture	*E. coli*	-	-	-	[141]
**19**	Nanoparticles on microstructured surface (capillary-driven detection) (antibody-mediated specificity)	Fe_3_O_4_ MNPs on microfluidic chip	PMMA at CNC machine for the microfluidic chamber. Two-seed growth method followed by chemical reduction method and self-assembly	785 nm	60 min	Milk	In four-step (chambered) microfluidic device, *E. coli*–MNP conjugates were injected Binding with *E. coli* antibodies and washing.	1080 cm^−1^, 1590 cm^−1^,	7 CFU/mL	-	[25]
**20**	Drop-casting and mixing	Fe_3_O_4_@PEI and MNComposite@ Au@Ag	Hydrothermal and chemical reduction followed by coating	785 nm	20 min	Fish pathogen *E. coli*	*E. coli* cells mixed and incubated with Fe_3_O_4_@PEI. Washed, drop-casted, and coated with Au@Ag NPs	537 cm^−1^, 564 cm^−1^, 652 cm^−1^, 730 cm^−1^, 792 cm^−1^, 955 cm^−1^, 1090 cm^−1^, 1330 cm^−1^, 1372 cm^−1^, 1456 cm^−1^, 1597 cm^−1^	10^5^ CFU/mL	-	[181]
**21**	Mixing and drop-casting	Fe_3_O_4_@AuNPs@NSP (nanoscale silicate platelets)	Coating on NSP sheet then Fe3O4 linking with coprecipitation method	632.8 nm	--	Bacterial culture	*E. coli*	734 cm^−1^, 1599 cm^−1^	10^3^ CFU/mL	3.4 × 10^6^	[190]
**22**	Nanoparticle coating on microstructure surface	Flexible plasmonic microneedles	Bioprinting/soft lithography followed by coating	785 nm	15 min	Mutton	Substrate pressing on Agar plates	715 cm^−1^, 960 cm^−1^, 1025 cm^−1^, 1140 cm^−1^, 1270 cm^−1^, 1457 cm^−1^, 1585 cm^−1^,	143 CFU/gm	1.2 × 10^4^	[29]
**23**	Drop-casting and mixing (antibody-mediated specificity)	Lectin-functionalised MNPs with covalent organic framework	Schiff base sequential method	785 nm	--	Bacterial culture	*E. coli* cells were mixed with MNPs, washed, centrifuged, and mixed with *antibody-modified* nanotags, incubated, washed, and drop-casted	2271 cm^−1^	10^1^ CFU/mL	-	[182]
**24**	Nanoparticle coating on microstructure surface	3D-ACEK/SERS system and AgNPs	Photo-lithography	532 nm	2 min	Whole blood	Blood sample was mixed with AgNPs and drop-casted inside the chamber	754 cm^−1^, 1102 cm^−1^, 1156 cm^−1^, 1204 cm^−1^, 1286 cm^−1^, 1362 cm^−1^, 1466 cm^−1^	3 CFU/mL	-	[213]
**25**	Mixing and drop-casting (affinity-mediated specificity)	Lectin (Con A)-modified BCNCs and AuNPs	Extraction, functionalisation, and mixing	785 nm	1 s	Bacterial culture	*E. coli* was added with lectin and bacterial cellulose nanocomposites (BCNCs) mixture. Mixed, washed, resuspended with AuNPs, and drop-casted	730 cm^−1^, 1000 cm^−1^, 1241 cm^−1^, 1326 cm^−1^, 1466 cm^−1^, 1560 cm^−1^	∼1.5 × 10^3^ CFU/mL	-	[182]
**26**	Nanoparticles coated on nanostructure surface	Aptamer-Au@MMSPM Microarray biochip	Frends method followed by self-assembly	785 nm	6 s	Bacterial culture, milk, and minced pork	Sample was incubated with Au@MMSPMs, washed, and mixed with nanotags, incubated and drop-casted	730 cm^−1^, 1050 cm^−1^, 1300 cm^−1^	2.20 CFU/mL	-	[183]
**27**	Mixing and drop-casting	AgNPs	Chemical reduction method	785 nm	15 s	Bacterial culture	*E. coli* cells were washed and mixed with AgNPs and drop-casted	450 cm^−1^, 472 cm^−1^, 627 cm^−1^, 706 cm^−1^, 850 cm^−1^, 930 cm^−1^, 1220 cm^−1^, 1310 cm^−1^	-	-	[210]
**28**	Microstructures of plasmonic material	Vertically aligned gold nanowires on silicon wafers	Seed growth method	785 nm	10 s	Ocular swab	Herringbone structure was used to fasten the PCR. Enriched bacterial samples were drop-casted on the v-AuNW	681–691 cm^−1^, 1021–1225 cm^−1^, 1182–1184 cm^−1^, 1269–1277 cm^−1^, 1517–1524 cm^−1^	-	3.16 × 10^−6^ M	[219]
**29**	Mixing and drop-casting	Self-supporting liquid-free membrane and halide-modified AgNPs with antibodies and indole	Chemical reduction method followed by self-assembly	633 nm	5 s	Bacterial culture	Sample was mixed and drop-casted	1063 cm^−1^	0.3 μM	-	[220]
**30**	Nanostructure modified with plasmonic material	ODPA-modified AuNPs/TiNTs	Electrochemical anodisation followed by sputtering and photoreduction	638 nm	5 s (20 min total detection time)	Bacterial culture	*E. coli* cells were drop-casted	2136 cm^−1^,	3 cells per mL	4.0 × 10^7^	[221]
**31**	Mixing and drop-casting (aptamer-based detection)	Au NP-based aptasensor	Chemical reduction method for NPs and freeze recovery method aptamers	633 nm	10 s (45 min total detection time)	Bacterial culture along with tap water, drinking water, and milk	Bacterial cells were mixed with the magnetic beads and ds-DNA and incubated. Ligase, polymerase (enzymes), and SERS probe (Au NPs) were drop-casted	1504 cm^−1^,	0.3 CFU/mL	-	[214]
**32**	Nanostructure surface with plasmonic material/nanoparticle coating	Ag-coated silicon nanowires	Wet-chemical MACE method	785 nm	10 s (total detection time 5 min for 10 samples)	Synthetic urine	Serial dilution and drop-casting	728–739 cm^−1^, 966–982 cm^−1^, 1101–1135 cm^−1^, 1183–1188 cm^−1^, 1280 cm^−1^, 1333 cm^−1^, 1147 cm^−1^, 1583–1588 cm^−1^,	100 CFU/mL	-	[208]
**33**	Mixing and drop-casting (drop-casting through an acoustic printer)	Au nanorods	Seed growth method	785 nm	3 min	Bacterial culture	Mixed samples were drop-casted/printed using the acoustic printer	735–744 cm^−1^, 1056–1090 cm^−1^, 1097–1099 cm^−1^, 1204–1209 cm^−1^, 1240–1248 cm^−1^, 1330–1335 cm^−1^, 1449–1450 cm^−1^, 1529–1534 cm^−1^,	-	1.5 × 10^2^	[146]
**34**	Drop-casting (antibiotic treatment)	AgNPs	Chemical reduction method	785 nm	-	Bacterial culture	Direct drop-casting of AgNPs on petri plate	-	-	-	[222]
**35**	Mixing and drop-casting (ML algorithms for rapid detection)	AgNPs	Chemical reduction method	784.56 nm	-	Bacterial culture	Single colony was mixed by vortexing in PBS buffer followed by mixing with AgNPs and drop-casting	530 cm^−1^, 656 cm^−1^, 730 cm^−1^, 1248 cm^−1^, 1320 cm^−1^, 1414 cm^−1^, 1450 cm^−1^, 1576 cm^−1^,	-	-	[184]
**36**	Nanostructure-based microfluidic chip	Functional organic polymer/Au nanofilm	Chemical reduction method	785 nm	30 s (total detection time 60 min)	Bacterial culture	Direct sample injection in microchannel	601 cm^−1^, 813 cm^−1^, 990 cm^−1^, 1280 cm^−1^, 1450 cm^−1^, 1610 cm^−1^, 1720 cm^−1^,	-	8.8 × 10^5^	[209]
**37**	Nanostructure surface coated with plasmonic materials (aptamer-based sensor)	SH-apt@Au-Ag@Si TP and ROX-aptamer	MACE method	785 nm	0.5 s (total detection time 210 min)	Bacterial culture (able to differentiate between the bacteria)	Drop-casting the bacteria on SH-apt@Au-Ag@Si TP followed by the ROX-aptamer drop-casting	663 cm^−1^, 735 cm^−1^, 1460 cm^−1^,	2.8 CFU/mL	1.53 × 10^7^	[171]
**38**	Mixing and drop-casting	AuNPs (compared the liq vs. drop-casting method)	Turkevich’s method	785 nm	20 s	Bacterial culture	Mixed and spectra taken Mixed and drop-casted and spectra taken (comparison)	663–664 cm^−1^, 730–732 cm^−1^, 954–960 cm^−1^, 1244–1246 cm^−1^, 1341–1348 cm^−1^, 1452 cm^−1^, 1690–1700 cm^−1^,	-	3.4 × 10^5^	[59]
**39**	Mixing and drop-casting (aptamer-based detection)	AuNPs	Chemical reduction method	785 nm	5 s	Bacterial culture, tap water, and milk	Aptamer was mixed and incubated with *E. coli* Centrifuged, mixed and incubated with SERS prob, then drop-casted	1073 cm^−1^, 1586 cm^−1^, 2227 cm^−1^,	409 CFU/mL	-	[223]
**40**	Flexible microstructure surface coated with nanoparticles	Ag NPs@PDMS sponge	Chemical reduction method	532 nm	1 s	Bacterial culture and milk	-	658 cm^−1^, 724–738 cm^−1^, 1242–1248 cm^−1^, 1330 cm^−1^, 1467 cm^−1^, 1622 cm^−1^,	1CFU/mL	1.53 × 10^8^	[200]
**41**	Nanoparticles coated on flexible substrate	Ti_3_C_2_Tx-AuNP-based paper substrates	Ti_3_C_2_T*_x_* synthesis, AuNPs synthesis and mixing by self-assembly followed by dip coating of cellulose filter paper	633 nm	60 s	Bacterial culture and porcine skin	*E. coli* cells were centrifuged, washed, and resuspended in NaCl solution. Filter paper-based substrate was dipped in bacterial suspension and dried	657 cm^−1^, 660 cm^−1^, 734–735 cm^−1^, 960–962 cm^−1^,	5 × 10^5^ CFU/mL	-	[191]
**42**	Mixing and drop-casting	Au–WS_2_ nanohybrids	Chemical reduction method and drop-casting on silicon wafer	780 nm	20 s	Bacterial culture	*E. coli* culture was diluted in PBS and drop-casted	736 cm^−1^, 1149 cm^−1^, 1210 cm^−1^, 1306 cm^−1^, 1438 cm^−1^, 1494 cm^−1^	10^4^ CFU/mL	∼1.80 × 10^9^	[196]
**43**	Mixing and drop-casting	2D-functionalised MoS_2_ and SnS_2_	Hydrothermal followed by self-assembly	633 nm	-	Bacterial culture	*E. coli* cells were washed and suspended in PBS, mixed with SERS substrate, and drop-casted	828 cm^−1^, 1129 cm^−1^, 1159 cm^−1^, 1240 cm^−1^, 1300 cm^−1^, 1499 cm^−1^,	-	-	[207]
**44**	Mixing and drop-casting	AgNPs	Chemical reduction method	785 nm	10 s	*E. coli* biofilm	Biofilm was mixed and incubated with AgNPs and drop-casted	653 cm^−1^, 726 cm^−1^, 955 cm^−1^, 1000 cm^−1^, 1128 cm^−1^, 1245 cm^−1^, 1318 cm^−1^,	-	-	[176]
**45**	Mixing and drop-casting on a 2-electrode surface	FF-PNT/Ag NP and Au electrodes on silicon sheet	Self-assembly and 3D printing (electric potential enhanced the SERS signals by 4–5 fold	532 nm (with 0–25 V potential difference, 5 V stepwise)	1 s	Bacterial culture and human blood	Sample was mixed with FE-PNT/Ag NPs and drop-casted	653 cm^−1^, 730 cm^−1^, 1001 cm^−1^, 1150 cm^−1^, 1330 cm^−1^, 1360 cm^−1^, 1665 cm^−1^,	10 CFU/mL	-	[177]
**46**	Mixing and drop-casting	MB@Ag@Au	Self-assembly	785 nm	10 s	Bacterial culture	Cells were drop-casted and dried on the MB@Ag@Au beads surface arranged on the surface of silicon wafer	651 cm^−1^, 727 cm^−1^, 1378 cm^−1^,	-	-	[224]
**47**	Microstructure surface coated with nanoparticles	Microcavity Array-Based Digital SERS Chip	Photolithography, etching and sputtering	-	-	Bacterial culture	Centrifuged, washed, resuspended in LB broth, and injected	733 cm^–1^, 876 cm^–1^, 918 cm^–1^, 1146 cm^–1^, 1260 cm^–1^ 1328 cm^–1^, 1450 cm^–1^	-	1.1 × 10^8^	[187]
**48**	Drop-casting	PEG-AuNPs@Al foil	Chemical reduction method	785 nm	10 s	Bacterial culture and urine	Centrifuged, washed, and drop-casted	-	10^5^ cells/mL	10^8^	[179]
**49**	Drop-casting	V_2_CTx-AuNSs@Silicon wafer	Electrostatic self-assembly	808 nm	-	Bacterial culture	Cells were washed 3 times and resuspended, incubated with V_2_CTx-AuNSs overnight and groupcasted on silicon wafer	733 cm^–1^, 753 cm^–1^,	1 × 10^5^ CFU/mL	-	[188]
**50**	Microstructure surface coated with nanoparticles	Au nano stars @AGMS (Array Gas membrane separation) device	Photo-driven synthesis	785 nm	2 s	Bacterial culture and Sour food	The culture was washed and kept in AGMS device for treatment; Au nanostars were also added	751 cm^–1^, (indole detection)	5 CFU/mL	-	[225]

## Data Availability

Not applicable.
